# Catalytic activity and mechanistic investigation of 1D 2-Picolinic acid based Cu(II) coordination polymer in the selective construction of 1,4-disubstituted triazoles

**DOI:** 10.1038/s41598-022-18780-x

**Published:** 2022-08-26

**Authors:** Merangmenla Aier, Firdaus Rahaman Gayen, Amrit Puzari

**Affiliations:** 1 Department of Chemistry, National Institute of Technology Nagaland, Chumoukedima, Dimapur, Nagaland 797103 India; 2 Advanced Materials Group, Materials Sciences and Technology Division, CSIR-North East Institute of Science and Technology, Jorhat, Assam 785006 India

**Keywords:** Chemistry, Materials science

## Abstract

The catalytic activity of 1D 2-Picolinic acid based Cu (II) coordination polymer (**CP1**) in click reaction was evaluated to generate 1,4-disubstituted 1,2,3-triazoles selectively. The **CP1** catalyst loading of 2 mol% was applied successfully in the reaction for primary azides with diverse functionalities of terminal alkynes in green solvent (EG/H2O). Moreover, the one-pot, multicomponent click reaction involving benzyl bromide, sodium azide, and phenylacetylene was also catalyzed by **CP1**. The findings show that 1D 2-Picolinic acid based Cu (II) coordination polymer catalytic systems are highly efficient for green click triazoles synthesis. DFT calculation supported the plausible mechanism involved in the **CP1** catalyzed click reaction.

## Introduction

1,2,3-Triazoles are a vital category of five-membered heterocycles that are increasingly found in diverse applications in chemistry, biology, medicine, and materials science^1–3^. Many 1,2,3-triazole compounds are clinically important and exhibit various biologically important activities such as antibiotic^4^, anti- Human Immunodeficiency Virus drug^5^, anticancer^6^, antiviral^7^, etc*.* As a result, there is immense interest in the emerging synthetic protocols for these heterocycles^8^. Huisgen 1,3-dipolar azide/alkyne cycloaddition is the conventional method for 1,2,3-triazoles synthesis. However, the classical thermal conditions which does not use a catalyst requires high temperature and long reaction time, which results in yielding a mixture of 1,4 and 1,5-substituted triazoles without regioselectivity^9^. The Cu-catalyzed Huisgen 1,3-dipolar azide/alkyne cycloaddition, popularly known as ‘Click reaction’, which was found independently by Sharpless^10^ and Meldal^11^ groups, is considered to be the most powerful approach for 1,2,3-Triazoles preparation. This transformation speeds up and enables the milder reaction conditions with a wide substrate scope and absolute regioselectivity (1, 4-substituted triazoles). Numerous researches have suggested that copper source which can create the catalytically active Cu(I) species in the reaction media can possibly initiate the click reaction. In general, the commonly used catalytic system mostly make an extensive use of Cu(II) salt and reducing agent (e.g. sodium ascorbate^12^ or hydrazine hydrate^13^) in water or mixture of water with an organic solvent. However, the difficulty in removing the copper ions from the synthesized products using the commonly practiced catalytic systems limits its application in pharmaceutical and biological sciences^14^. To overcome this problem, many new alternative catalytic materials have been developed by various researchers over the past years. An iron based Cu nanoparticles catalytic system for click reaction was developed by Lipshutz and coworkers^15^. A nano Cu-catalyst in polydiacetylene micelles was reported by Doris et al.^16^ for Huisgen cycloaddition reaction. Recently, a nanofibrous copper (0) catalytic system for azide/alkyne cycloaddition reaction was reported by Sayyahi et al.^17^. In 2019, Esmaeilpour et al.^18^ have introduced a reusable Fe_3_O_4_@SiO_2_-dendrimer-encapsulated Cu(II) catalyst for CuAAC reaction. In spite of these considerable achievements from various research studies in the recent past, most of the present systems of catalysis have limitations for practical applications because of the requirement for expensive additive, organic solvent, high catalyst loading and long reaction time. Therefore, it has become indispensable to develop new systems of CuAAC reaction which is efficient, economical, greener, and low Cu-loading.

In the recent decades, coordination polymers (CPs) that are formed from organic bridging ligands with transition metal ions have evolved as one of the fastest emerging area of chemical studies because of their remarkable structural features and distinctive functionalities^19^. Gas storage^20,21^, drug delivery^22,23^, photocatalysis^24,25^, magnetism^26,27^, luminescence^28,29^, and catalysis^30,31^ are some of the uses of these hybrid materials. Numerous catalytic studies on the potential uses of 2D and 3D CPs have been reported in reactions like Knoevenagel reaction^32,33^, oxidation^34,35^, photocatalysis^36^, Heck reaction^37^, and Click reaction^38^. However, the potential applications of one-dimensional CPs were given lesser attention. Despite their structural simplicity, the 1D CPs are comparatively much easier to synthesize and their topological structures can be tuned more effectively to maximize the scope in different applications^39,40^. Thus, more effort should be made to systematically study the catalytic application of 1D CPs. The carboxylate ligands are usually employed in designing the CPs because of their diverse coordination modes and bridging ability. Copper(II) carboxylates, especially those with nitrogen donor ligands, have been extensively investigated because of their intriguing topological structures and properties.

In our recent publication^41^ the structural characteristics and photocatalytic activity of 1D 2-picolinic acid based Cu (II) coordination polymer formulated as {[Cu(2-pic)_2_]2H_2_O}_n_ (**CP1**) (2-pic = pyridine-2-carboxylic acid) has been reported. To the best of our knowledge **CP1** has not been explored for catalytic application in organic synthesis. Intrigued by its interesting structures, and properties and also considering the need to develop new catalytic system for CuAAC reaction with low Cu-loading, economical, and greener, we took interest in investigating the catalytic property of **CP1** in the synthesis of 1,2,3-triazoles. This study presents a sustainable chemical protocol for the “click reaction” by employing **CP1** as the catalyst that can selectively provides 1,4-substituted triazole, instead of 1,5-substituted triazole. The reaction mechanism was further elucidated by DFT study. The catalytic property of **CP1** system under the influence of various parameters has also been investigated. The successful synthesis of one-pot multicomponent cycloaddition of benzyl bromide, phenylacetylene, and sodium azide adds further advantage to this catalytic system.

## Experimental section

### Materials and methods

All the chemicals used in this study were procured from Sigma-Aldrich/TCI, Japan and organic solvents were distilled in accordance with the standard procedure as required. The reactions performed in the present study were carried in oven-dried round-bottom flask in open air. Thin-layer chromatography was used to monitor progress of all the reactions by utilizing silica-gel 60F_254_ plates, which were then visualized under UV light (λ = 254 nm). Column chromatography method was used for purification of the products using silica gel (60–120 mesh). FTIR spectra of all the isolated 1,2,3-Triazole products were recorded on Agilent Cary 630 FTIR spectrometer. ^1^H NMR (500 MHz), and ^13^C NMR (125 MHz) were scanned on Bruker Avance III 500 MHz FT-NMR spectrometer in CDCl_3_ taking TMS as internal standard. Chemical shifts (δ) are shown in ppm, and the values of coupling constants (J) are expressed in hertz. Splitting patterns are denoted by the following abbreviations: s/singlet, d /doublet, t/triplet, q/quartet, and m/multiplet.

### Synthesis of CP1

The **CP1** was prepared following the same procedure as reported in our previous work^41^. A solution of Picolinic acid (0.246 g, 0.002 ml) in 5 ml of 1NHCl was added to a stirred solution of CuSO_4_.5H_2_O (0.498 g, 0.002 mol) in 5 ml of 1NHCl. After stirring for 30 min at 70 °C the blue solution was let to cool in ice water which resulted in the formation of dark blue crystals. The crystals were filtered and then recrystallized with ethanol, and finally dried at 60 °C in oven for 40 min to produce 40% Yield. *Anal. Calc.* for [C_12_H_8_N_2_O_4_Cu]0.2H_2_O: C, 41.92; H, 3.49; N, 8.15. *Found*: C, 41.25; H, 3.02; N, 8.18.

### General procedure for the 1D 2-Picolinic acid based Cu (II) coordination polymer (CP1) catalyzed azide-alkyne cycloaddition reaction 3a-n

Mixture for the reaction was prepared in a 50 ml round-bottom flask using azide (1.0 mmol), alkyne (1.2 mmol), sodium ascorbate (3 mol%), **CP1** (2 mol%), and 2 ml EG/H_2_O (1:1), and was stirred at 30 °C for 4 h. TLC was used in monitoring the reaction’s progress, and upon completion the mixture was extracted with (4 × 10 ml) ethyl acetate. Na_2_SO_4_ anhydrous was used for drying the organic layer, which was then concentrated under reduced pressure, and subsequently purified using column chromatography (n-hexane/ethyl acetate) to provide the targeted product 3a-3n (for details see SI).

1-Benzyl-4-phenyl-1*H*-1,2,3-triazole (**3a**): White solid; Yield; IR (ν_max_ cm^−1^): 3126, 3059, 2923, 1607, 1464 (CH_2_), 1440, 1425, 1355, 1222 (N–N = N-), 1194 (C-N), 1073, 1047, 974, 912, 825 (= C-H oop, triazole ring), 763, 725, 691, 578. ^1^H NMR (400 MHz, CDCl_3_): δ = 7.81 (d, J = 7.2 Hz, 2H), 7.66 (s, 1H), 7.42–7.36 (m, 5H), 7.34–7.29 (m, 3H), 5.58 (s, 2H). ^13^C NMR (101 MHz, CDCl_3_): δ = 148.33, 134.75, 130.60, 129.26, 128.89, 128.26, 125.78, 119.55, 54.33. HRMS (ESI) calcd for C_15_H_13_N_3_ [M + H]^+^: 236.1109, found: 236.1186.

### General procedure for the 1D 2-Picolinic acid based Cu (II) coordination polymer (CP1) catalyzed one-pot azide-alkyne cycloaddition reaction

Benzyl bromide (1 mmol), sodium azide (1.1 mmol), phenylacetylene ( 1.2 mmol), 3 mol% of sodium ascorbate (3 mol%), 2 mol% of **CP1** (2 mol%), and 4 ml of EG/H_2_O (1:1) were mixed in a 50 ml RB flask, and was stirred at 30 °C for 4 h. TLC was used in monitoring the reaction’s progress, and upon completion the mixture was extracted 4 times with ethyl acetate. Na_2_SO_4_ anhydrous was used to dry the organic layer, which further was concentrated under reduced pressure, and subsequently purified using column chromatography (n-hexane/ethyl acetate) to provide the 1-benzyl-4-phenyl-1H-1,2,3-triazole.

### X-ray crystallography

The SCXRD data of 1D Copper-based coordination polymer (**CP1**) were generated using Bruker Nonius SMART APEX II diffractometer equipped with a Charge-Coupled Device (CCD) area detector and graphite-monochromated Mo Kα radiation (λ = 0.71073 Å) operating at 296 K. Crystal structure refinement was performed with SHELXL-14/7 program^42^ using the full-matrix least-squares method on F2. All the non-hydrogen atoms of **CP1** were refined anisotropically against F2 of all reflections. The H-atoms of the organic ligands were placed at their geometric positions and refined isotropically. CCDC 2,094,267 contains the crystallographic data for **CP1**.

### Computational details

The geometry optimization for all the interested compounds, including the intermediates and transition states, was carried out in the gas phase using the DFT computations with B3LYP/631G(d, p)^43,44^ basis set in Gaussian 09 packages^45^. LANL2DZ basis set was employed to describe the Cu atom. Frequency calculations were executed to analyze the stationary points (zero imaginary frequency)/ transition states (one imaginary frequency) at the identical theoretical level. The effects of solvation or dispersion forces were not taken into account in our calculations because they probably differ from those observed in real compounds. Literature on the theoretical features of CuAAC reaction mechanism suggested that these effects do not seem to be the critical parameters/characteristics for those general reactions that work experimentally for a family of reactive compounds. The Gibbs free energy at 298.15 k was calculated using frequency calculations. The TS was optimized applying Berny algorithm (opt = ts) at the identical theoretical level.

## Results and discussion

We initiated our investigation of 1D Copper (II) coordination polymer-catalyzed cycloaddition using benzyl azide and phenyl acetylene as the model substrates. The reaction was tested in presence of **CP1** (2 mol%) as homogeneous catalyst, Na ascorbate (3 mol%) in water under open-air condition at 30 °C, which led to 60% yield of 1-benzyl-4-phenyl-1*H*-1,2,3-triazole after 4 h (Table 1, entry 1). Encouraged by the exciting transformation, we have screened the efficacy of different solvents with **CP1** (2 mol%) and Na ascorbate (3 mol%) in the CuAAC cycloaddition reaction. The reaction proceeded in aprotic solvents like DMF, CH_3_CN, DMSO, and THF, but resulted in inferior yields (entries 2–5). On the contrary, the reaction proceeded well with protic solvent MeOH and resulted in a 53% yield of the product **3a** (entry 6). It’s worth noting that when the reaction was tested in ethylene glycol, the yield increased significantly (entry 7). On further evaluation it was found that mixed solvents of EG/H_2_O gave the best activity with considerable increase in the yield (entry 10). With EG/H_2_O as the optimal solvent we proceeded to examine catalyst loading for the reaction and discovered that lowering the catalyst loading to 0.5% leads to a decline in the conversion (entries 11, 12). It was also found that there was no significant improvement in the yield of the corresponding 1,4-disubstituted 1,2,3-triazole by increasing the catalyst loading to 3%, (entries 13, 14). The reaction failed in the absence of **CP1** (entry 15). We also investigated the reaction in absence of Na ascorbate and the product conversion was found to be in trace amount only (entry 16). Therefore, the reaction conditions in entry 10 have been evaluated to be the most suitable one for establishing a method which has broader application with lower reaction times. These findings suggested that strong reactivities in these catalytic systems required both **CP1** and Na ascorbate.

**Table 1:** Catalyst and Solvent optimization studies^a^.


Entry	Catalyst	Catalyst (mol%)	Na Ascorbate (mol%)	Solvent	Yield^b^ (%)
1	CP1	2.0	3.0	H_2_O	60
2	CP1	2.0	3.0	DMF	20
3	CP1	2.0	3.0	THF	15
4	CP1	2.0	3.0	CH_3_CN	10
5	CP1	2.0	3.0	DMSO	12
6	CP1	2.0	3.0	MeOH	53
7	CP1	2.0	3.0	EG	79
8	CP1	2.0	3.0	MeOH/H_2_O	65
9	CP1	2.0	3.0	THF/H_2_O	40
10	CP1	2.0	3.0	EG/H_2_O	86
11	CP1	1.0	3.0	EG/H_2_O	83
12	CP1	0.5	3.0	EG/H_2_O	83
13	CP1	2.5	3.0	EG/H_2_O	86
14	CP1	3.0	3.0	EG/H_2_O	87
15	CP1	0.0	3.0	EG/H_2_O	0
16	CP1	2.0	0.0	EG/H_2_O	Trace
17	CuSO_4_.5H_2_O	2.0	3.0	EG/H_2_O	69
18	Cu(II)complex	2.0	3.0	EG/H_2_O	40

^a^Reaction conditions: phenylacetylene (0.6 mmol), benzyl azide (0.5 mmol), **CP1** (0–3 mol%), Na ascorbate (0–3 mol%), solvent (2 ml); ^b^Isolated yields; Cu(II) complex: chloro glycinato 1,10-phenanthroline Cu(II) monohydrate.

We have also screened other copper catalysts under the same reaction conditions, and the optimization studies have shown that moderate yields of the product were formed when CuSO_4_.5H_2_O and Cu(II) complex were used as catalyst (entries 17–18). This result suggests the significance of **CP1** in promoting CuAAC reactions.

On successful establishment of the optimal reaction conditions, the scope of the catalyst method was tested with a variety of alkynes and azides. All the reactions were regioselective for 1,4-disubstituted triazoles and took 3–5 h to complete. The treatment of **1a** with phenylacetylenes consisting of electron-donating substituents (4-OMe, 3-Me) proceeded effectively to afford the corresponding 1,4-disubstituted-1,2,3-triazole in 61–88% isolated yields (Table 2, 3c–3f). However, the electron-poor (4-CF_3_, 4-F) aromatic alkynes on reaction with **1a** shows a negative influence providing 50–64% isolated yields of the 1,4-disubstituted-1,2,3-triazole products (Table 2, 3g–3i). We found that under the optimized conditions, the reaction between azides and heterocyclic alkyne such as 2-ethynylthiophene and 3-ethynylpyridine produces the desired triazole products with 85–90% yield (Table 2, 3j–3l). In addition, methyl propiolate also responded readily with azides under the optimized condition and gives high yield of the corresponding triazole products (Table 2, 3m–3n).

**Table 2:**
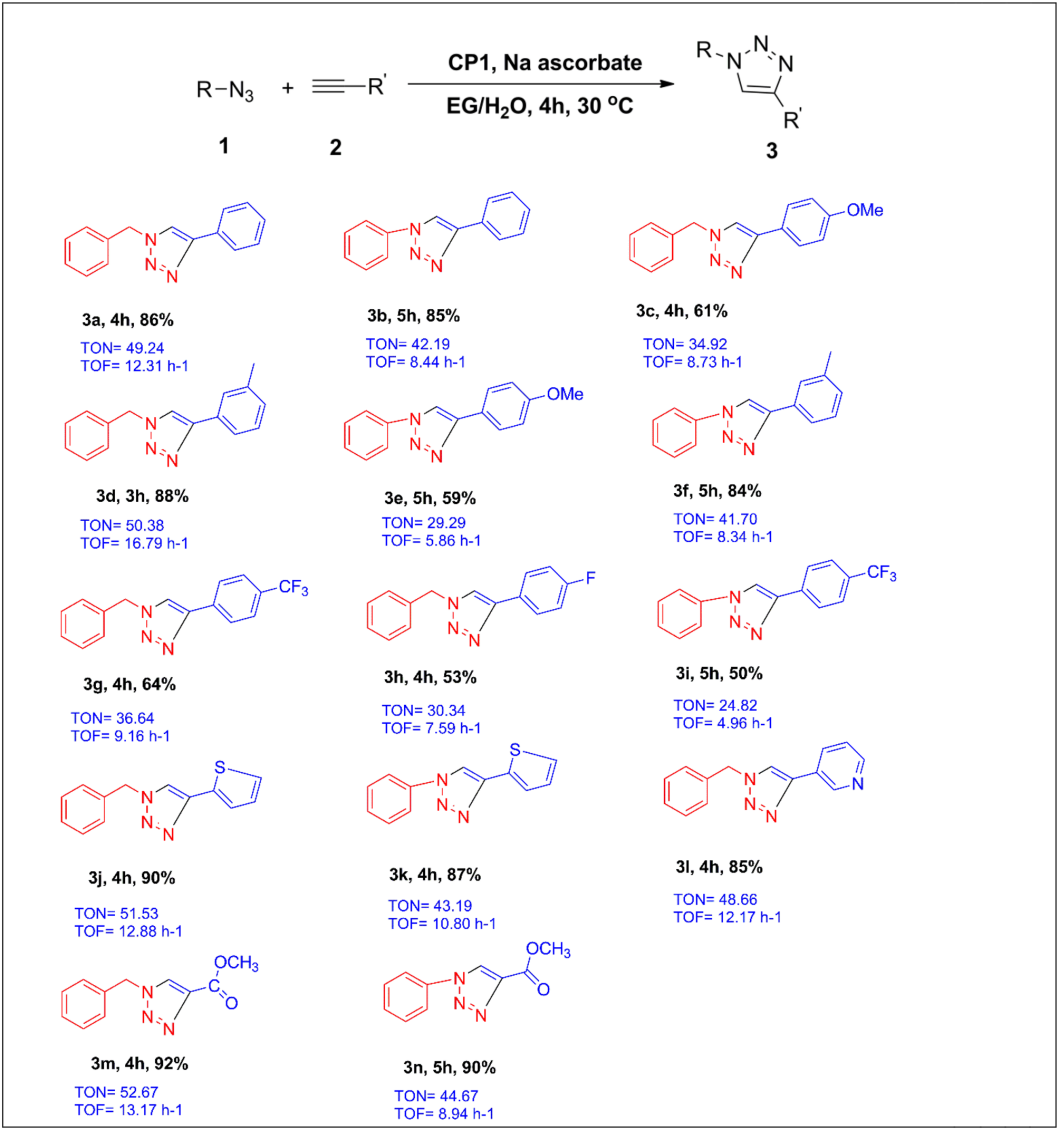
Substrate scope on 1D copper-based coordination polymer catalyzed azide/alkyne cycloaddition reaction^a^.

^a^Reaction conditions: azide (1.0 mmol), alkyne (1.2 mmol), **CP1** (2 mol%), Na ascorbate (3 mol%), 4 ml EG/H_2_O (1:1) at 30 °C in an open air. Yield of the products after column chromatography are mentioned.

Organic azides are generally safe and stable with water and oxygen but low molecular weight azides can be particularly hazardous to handle^46^. As a result, various methodologies have been developed for safe handling and isolation of organic azides in (3 + 2) cycloaddition reactions. We also examined our catalytic system for the one-pot, multi-component CuAAC reaction involving in situ formation of organic azide from aryl bromide with NaN_3_. Benzyl bromide was used as the precursor since the benzyl azide utilized in this study was prepared from the corresponding bromide. The **CP1** catalyst worked effectively for the multicomponent model reaction of benzyl bromide, NaN_3_, and phenylacetylene at the optimized reaction conditions, and generated the desired triazole product with high yield (Table 3, 4a). The one-pot reaction of benzyl bromide in presence of NaN_3_ with different alkynes proceeded satisfactorily to generate a good yield of 1,4-disubstituted-1,2,3-triazoles (Table 3, 4b–4d). The above outcomes show the potential benefit of a 1D Copper (II) coordination polymer catalyzed (3 + 2) cycloaddition reaction.

**Table 3:**
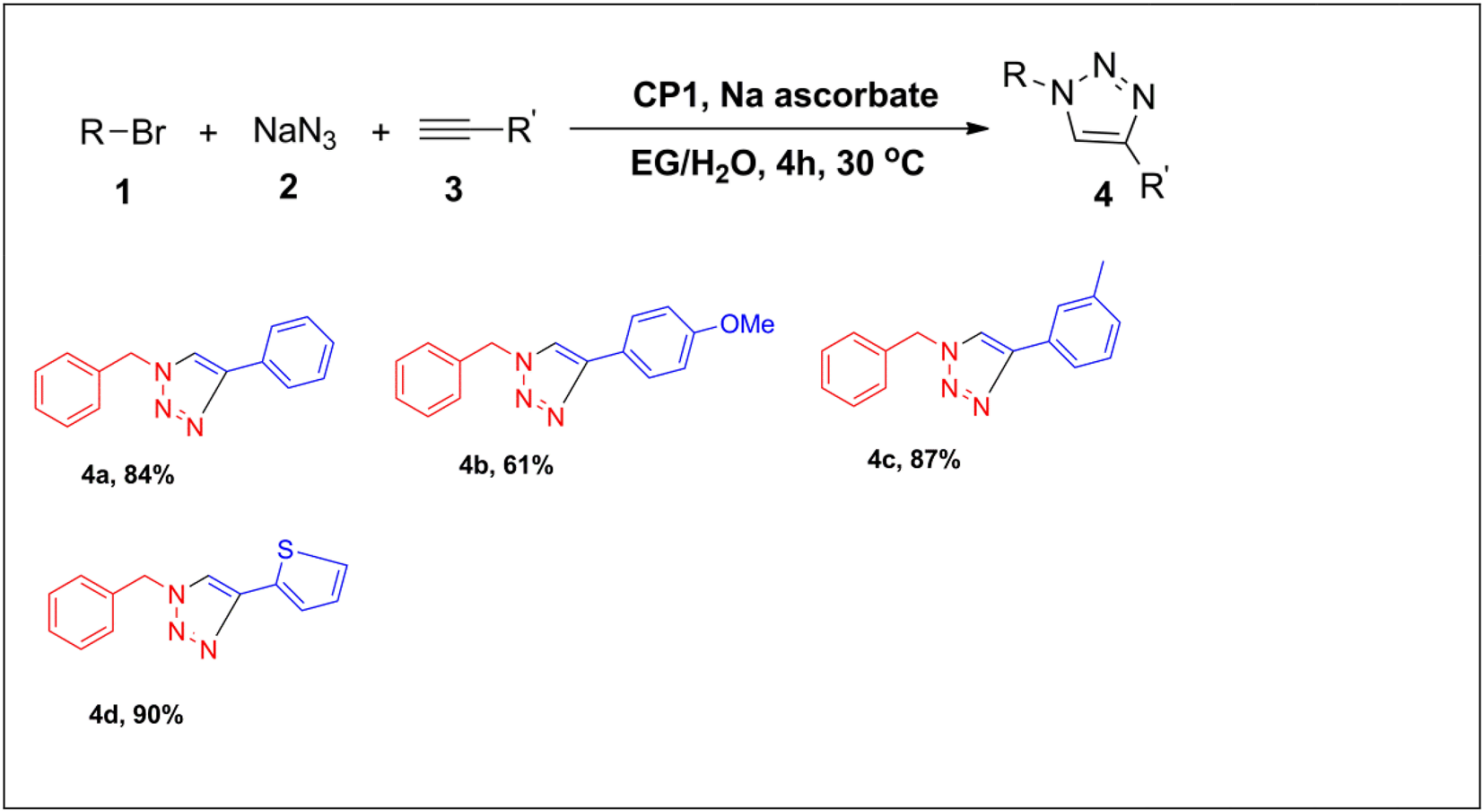
1D copper-based coordination polymer catalyzed multicomponent synthesis of 1,4-disubsttuted 1,2,3-triazoles^a^.

^a^Reaction conditions: Benzyl bromide (1.0 mmol), NaN_3_ (1.1 mmol), alkyne (1.2 mmol), **CP1** (2 mol%), Na ascorbate (3 mol%), 4 ml EG/H_2_O (1:1) at 30 °C in an open air. Yield of the products after column chromatography are mentioned.

**Figure 1:**
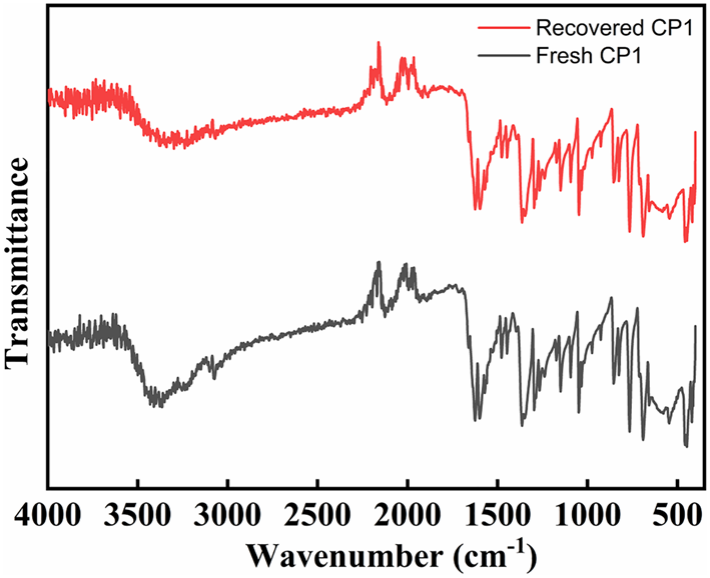
FTIR spectra of fresh and recovered **CP1**.

We then examined the possibility of recycling **CP1** homogeneous catalyst. The recycling experiment was performed with higher substrate concentration in order to avoid loss of material. The reaction employed phenylacetylene (4.8 mmol), benzyl azide (4.0 mmol) and Na ascorbate (3 mol%) in the presence of **CP1 (**2 mol%) in EG/H_2_O (16 ml) for 4 h. After the completion of the reaction, ethyl acetate was added to the reaction mixture to extract the product. The organic layer having the product and the EG/H_2_O layer having the homogeneous catalyst **CP1** were then separated. For the reusability study, the isolated EG/H_2_O layer was reemployed with addition of Molar quantities of reactants, and the experiment was allowed to proceed under the same reaction conditions. The first recycle reaction yielded 84% product (Table 4, entry 2). The experiment was repeated following the same procedure by separating the EG/H_2_O layer containing **CP1** catalyst and reused it in the subsequent reaction. A yield of 80% product formation (Table 4, entry 3) was observed in the second recycle reaction. This study shows that the **CP1** catalyst is able to retain its catalytic efficiency and maintain its selectivity towards the 1,4‐disubstitued 1,2,3‐triazole even after two recycle reactions. The stability of **CP1** catalyst was investigated after the second reaction recycle. The EG/H_2_O layer containing **CP1** catalyst was evaporated at 110 °C in an oven for 24 h, and then washed with ethanol. The **CP1** catalyst was recovered in solid form from the catalytic cycle. The FT-IR spectrum of **CP1** after 3 cycles as shown in Fig. 1 resembles that of the fresh **CP1** catalyst. Hence, it can be concluded that the **CP1** catalyst remains stable throughout the reaction process and is recyclable.

**Table 4:** Recyclability study of **CP1** catalyst.

Entry		Run		Yield^b^ (%)
1		First		86
2		Second		84
3		Third		80

**Figure 2:**
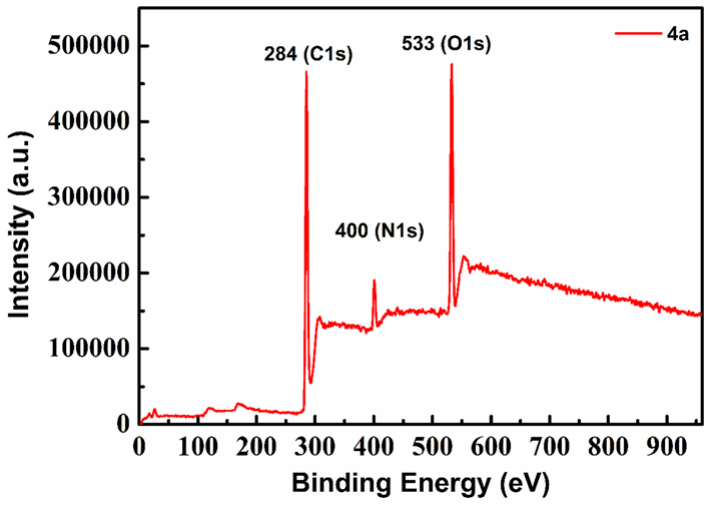
XPS survey spectrum of **4a**.

Further, in order to determine the Copper content in the extracted organic layer from the first run, ICP-MS analysis was performed. The result showed the presence of 0.00001 Wt % of trace Copper, which is negligible. The product was further isolated from the organic layer through solvent evaporation under reduced pressure followed by purification using column chromatography. The isolated product, as confirmed by XPS analysis, was free from Copper. As can be seen in the survey spectrum of the isolated product **4a** (Fig. 2), peaks corresponding to C, N and O were present but no such corresponding peak was detected for Cu, which indicated the absence of Copper in the isolated product.

The advantage of this method is that the product and catalyst can be isolated easily through a simple operation. Also, the solution containing the catalyst can be reused without the need to isolate the catalyst, and thereby reduces the operation cost and environmental hazards.

In order to understand the efficiency of our catalytic system, a comparative study with other reported catalytic system in copper-catalyzed reaction of benzyl azide and phenylacetylene to afford 1,4‐disubstituted 1,2,3‐triazoles are summarized in Table 5. Although each of these methods has their own advantages, they also have some short-comings including expensive catalyst synthesis, long reaction time, high catalyst loading and non-recyclable catalyst. As can be seen from the above discussion, our protocol is sustainable and has advantageous due to its operational simplicity such as ease of preparation of the **CP1** catalyst, easy separation process from the reaction mixture, and recyclability of the catalyst.

**Table 5:** Comparison of **CP1** catalytic protocols with other catalytic system in the synthesis of 1,4-disubstituted 1,2,3-triazoles.

Entry	Catalyst	Conditions	Time (h)	Yield (%)	Ref
1	Cu(II)-benzotriazole, 5 mol%	EtOH, 78 °C	24	93	^47^
2	GO-Cu(II)L^a^, 50 mg	*t*-Butanol/H_2_O, r.t, Na ascorbate (10 mg)	1	91	^12^
3	CuSO_4_-PEG-PS^b^, 5 mol%	H_2_O, N_2_, r.t., Na ascorbate (10 mol%)	12	97	^48^
4	Cu(II)-AHG^c^, 2 mol%	H_2_O, r.t	24	95	^49^
5	Cu(II)-polyethylenimine, 5 mol%	H_2_O, r.t	24	98	^50^
6	CuSO_4_, 10 mol%	*t*-BuOH/H_2_O, 65 °C, Na ascorbate (0.5 mmol)	12	82	^51^
7	Fe/Cu NPs, 1000 ppm Cu)	Et_3_N (0.5 equiv.), TPGS-750-M/H_2_O, r.t	6	99	^15^
8	Cu_2_O@pDAPEG, 0.35 mol%	H_2_O, r.t	24	99	^16^
9	Nano-porous Copper, 50 mg	H_2_O, 80 °C	6	92	^17^
10	Fe_3_O_4_@SiO_2_-dendrimer-Cu(II), 0.5 mol%	H_2_O, r.t	3	93	^18^
11	**CP1**, 2 mol%	EG/H_2_O, 30 °C, Na ascorbate (3 mol%)	4	86	This work

^a^Cu(II)L: Copper(II) *Bis*(2,20-bipyridine). ^b^PEG-PS: poly(ethylene glycol)-polystyrene.

^c^Cu(II)-alginate hydrogel, pDAPEG: polydiacetylene micelles-polyethyleneglycol.

### Mechanism

**Figure 3:**
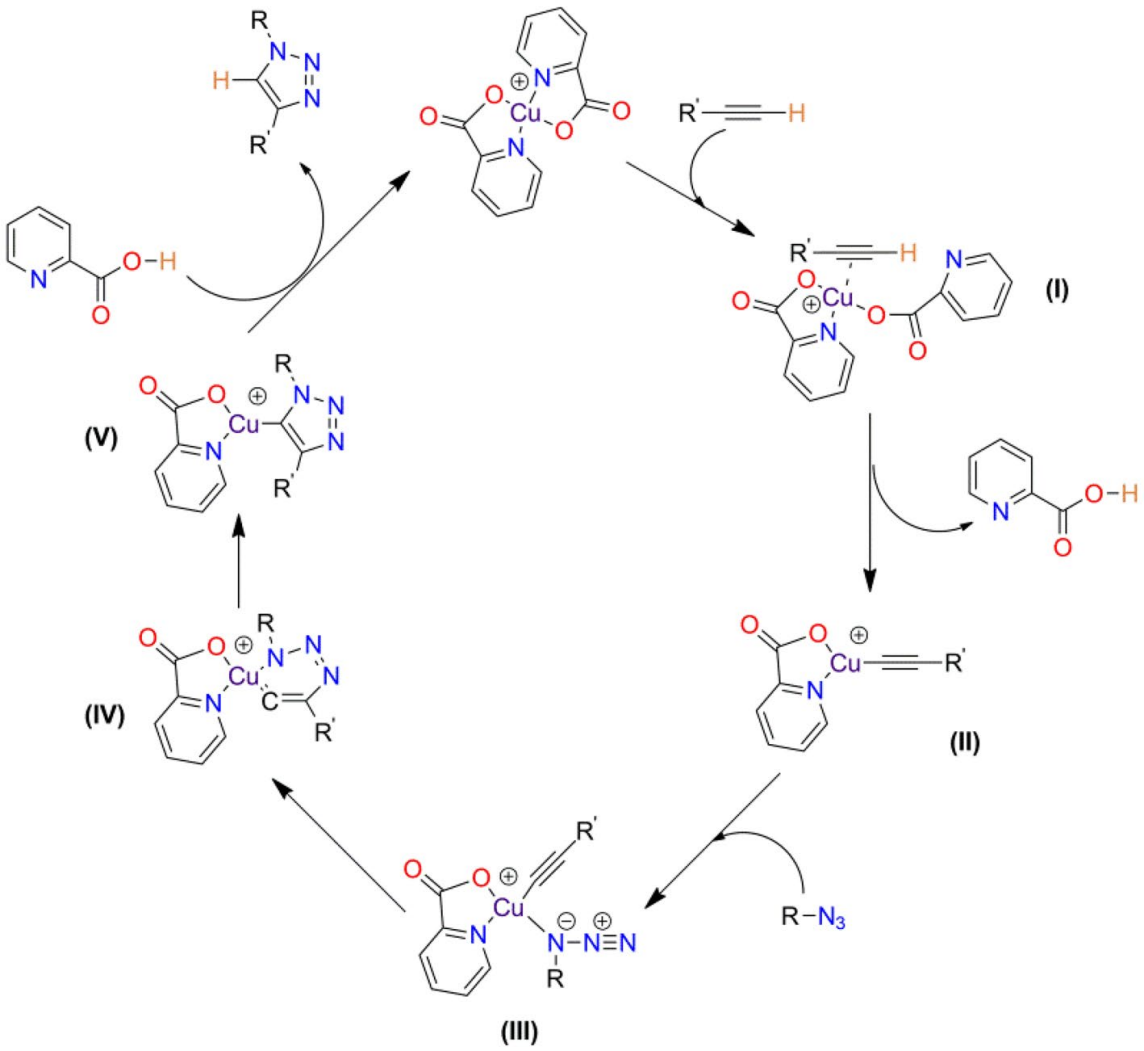
Proposed reaction mechanism of **CP1** in CuAAc.

The probable reaction mechanism involving **CP1** catalyst for the synthesis of 1,4-disubstituted triazoles is shown in Fig. 3^52^. When **CP1** is reduced to Cu(I) with sodium ascorbate, the reaction can be activated by coordinating the alkyne with the cationic Cu(I) center in a η2 mode. The –CH group in the alkyne moiety gets deprotonated by one of the copper coordinated picolinic acid ligands, resulting in the creation of a Cu(I)–acetylide complex. The organic azide further coordinates with the cationic Cu(I)–acetylide complex and transforms into a six-membered ring metallo-cycle, followed by contraction of the six-membered ring to produce a copper–triazolide (Fig. 3, Step V). The copper- triazolide acquires a proton from the picolinic acid ligands releasing the desired 1,4-disubstituted-1,2,3- triazoles, while regenerating the active catalyst to accomplish the cycle.

### DFT studies

**Figure 4:**
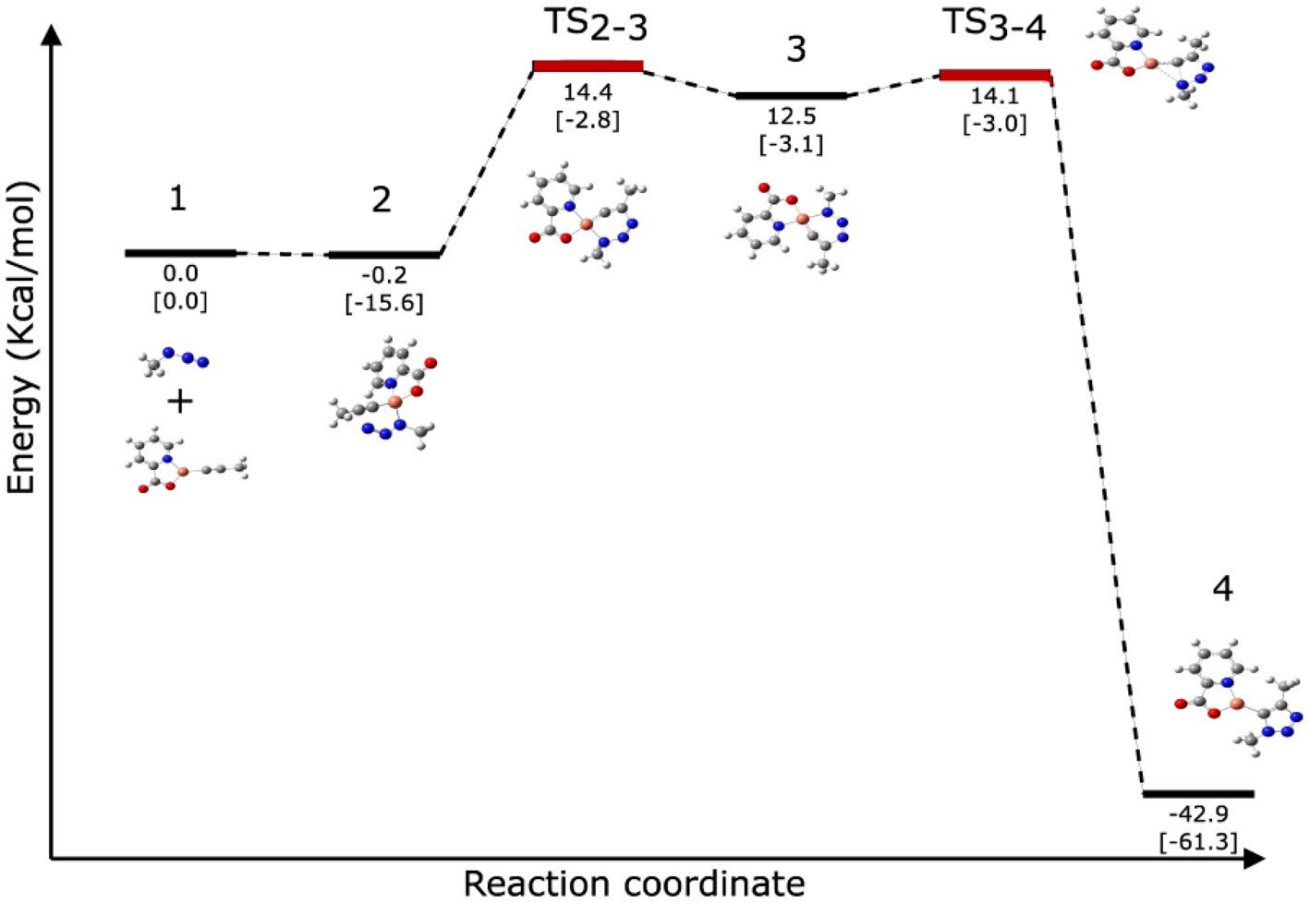
Calculated energy profile for the proposed reaction mechanism of **CP1** in CuAAC. Electronic energies (in round brackets) and free energies are shown in kcal/mol.

**Figure 5:**
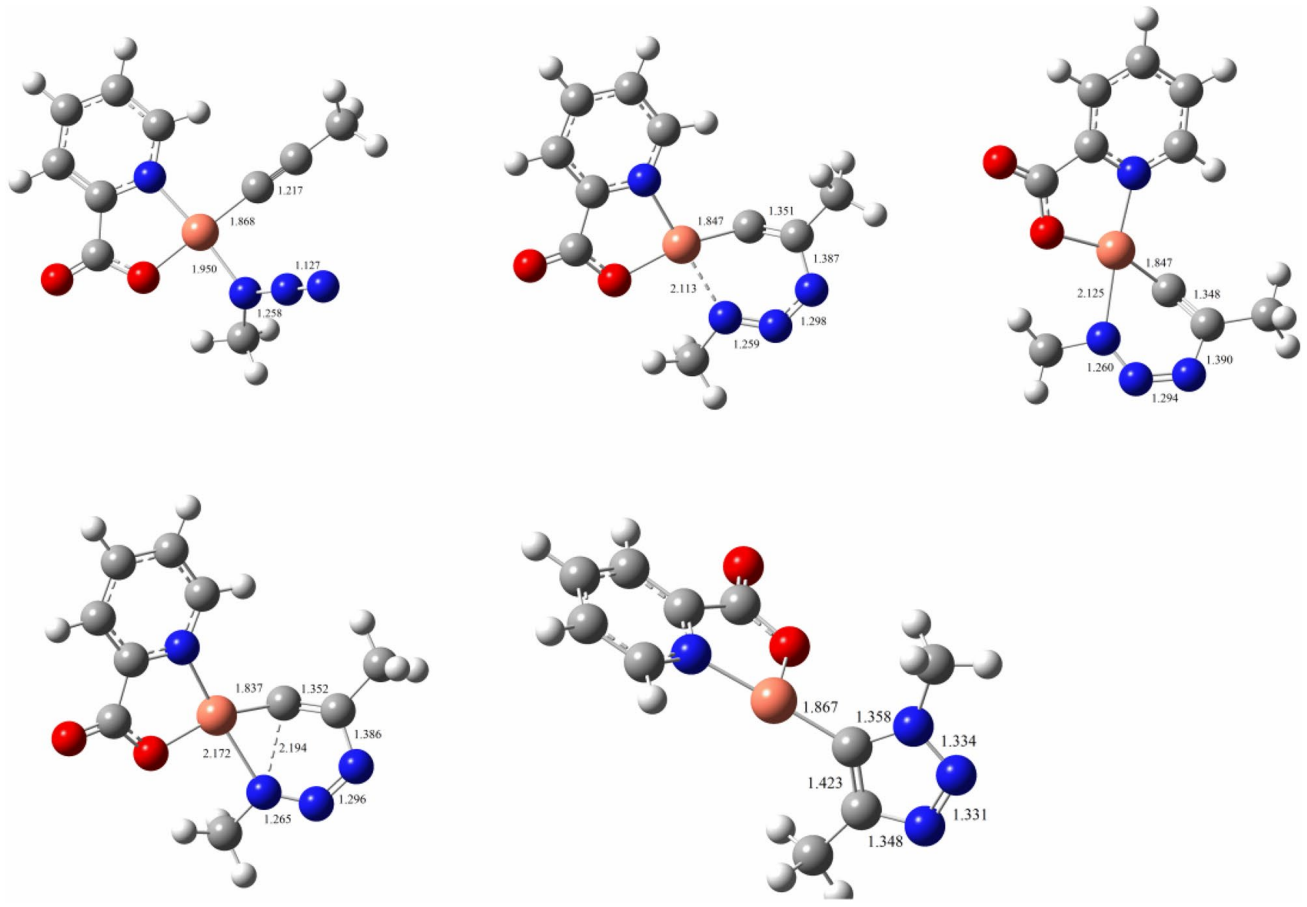
Optimized structures of TS_2/3_, TS_3/4_, and intermediates.

We have theoretically evaluated our hypothetical mechanism based on the proposed mechanism using the DFT calculations on a simplified system constituted of **CP1**, methyl azide, and propyne. The energy integrated with the suggested mechanism that provides the experimentally observed 1,4-triazole products were examined. In the initial step of the mechanism, propyne coordinates to the reduced Cu(I) centre by displacing one of the picolinic acid ligand resulting in a η^2^-alkyne complex. This Cu-acetylide complex was used as an initial reactant in Fig. 4. In the 2nd step, the methyl azide forms an azide-coordinated complex (**2**) by coordinating to the metal centre through the methylated nitrogen atom. The reaction of methyl azide with η^2^-alkyne complex to generate **2** was calculated to be slightly exothermic by 0.2 kcal/mol. Thereafter, the azide’s distal nitrogen in **2** binds the acetylide’s C-2 carbon creating the six-membered metallocycle **3**. This phase is endothermic and the computed barrier is 14.4 kcal/mol, which is also much lower than the reported mononuclear copper acetylide barrier (17 kcal/mol)^53^. The ring contraction barrier, from **3**, which forms the copper-triazolide complex **4** via TS_3/4_ is 14.1 kcal/mol. TS_3/4_ is 0.3 kcal/mol lower than TS_2/3_. The final step involves a rapid protonation of the copper-triazolide, which results in the triazole product; in the meantime, **CP1** active catalyst regenerates to complete the catalytic cycle. The geometries of the optimized intermediates, TS_2/3_, and TS_3/4_ are given in Fig. 5. The computed results suggest that the reaction pathway presented in Fig. 3 is favourable.

## Conclusion

In conclusion, we have employed a 1D Pyridine-2-carboxylic acid based Cu (II) coordination polymer as the active catalyst in green click triazoles synthesis with **CP1** catalyst loading of 2 mol%. The one-pot, multicomponent cycloaddition involving benzyl bromide, sodium azide, and phenylacetylene was also efficiently catalyzed by **CP1**. At low temperature (30 °C) the **CP1** catalytic system was adequately applied in selective construction of 1,4-disubstituted triazoles. The results suggest that **CP1**, which is simple to prepare, reusable and has a high substrate tolerance, can make considerable contribution to an extensive range of applications. Moreover, the DFT calculations were performed to support the experimental results of the proposed mechanism.

## Supplementary Information

Supplementary Information.

## Data Availability

All data are available in the main text or the supplementary materials. The datasets generated and/or analysed during the current study are available in the Cambridge Crystallographic Data Centre (CCDC 2,094,267) repository. https://www.ccdc.cam.ac.uk/structures/Search?Ccdcid=2094267&DatabaseToSearch=Published.
