# Supplementary material for: Catalytic activity and mechanistic investigation of 1D 2-Picolinic acid based Cu(II) coordination polymer in the selective construction of 1,4-disubstituted triazoles
Source: Sci Rep. 2022 Aug 26;12:14613. doi: 10.1038/s41598-022-18780-x (PMC9418271; doi:10.1038/s41598-022-18780-x)
Supplement: Supplementary file 1 — Supplementary Information. [file 41598_2022_18780_MOESM1_ESM.docx]

**Supporting Information**

**Catalytic activity and mechanistic investigation of 1D 2-Picolinic acid based Cu(II) coordination polymer in the selective construction of 1,4-disubstituted triazoles.**

Merangmenla Aier^a^, Firdaus Rahaman Gayen^b^ and Amrit Puzari^a^*

^a^Department of Chemistry, National Institute of Technology Nagaland, Chumoukedima, Dimapur, Nagaland, India-797103

^b^Advanced Materials Group, Materials Sciences and Technology Division, CSIR-North East Institute of Science and Technology, Jorhat, Assam-785006, India.

*E-mail: [amrit09us@yahoo.com](mailto:amrit09us@yahoo.com)

**Table of Contents**

Page

1. Characterization data of 1,4-disubstituted 1,2,3-triazoles………………… **S1-S4**
2. ^1^H NMR and ^13^C NMR Spectra of 1,4-disubstituted 1,2,3-triazoles……… **S4-S18**
3. IR Spectra of 1,4-disubstituted 1,2,3-triazoles………………………………**S18-S24**
4. Mass spectra of 1,4-disubstituted 1,2,3-triazoles……………………………**S25-S31**
5. References……………………………………………………………………**S31**
6. **Characterization data of 1,4-disubstituted 1,2,3-triazoles**





1-Benzyl-4-phenyl-1*H*-1,2,3-triazole (**3a**): White solid; Yield 86%; IR (ν_max_ cm^−1^): 3126, 3059, 2923, 1607, 1464 (CH_2_), 1440, 1425, 1355, 1222 (N-N=N-), 1194 (C-N), 1073, 1047, 974, 912, 825 (=C-H oop, triazole ring), 763, 725, 691, 578. ^1^H NMR (400 MHz, CDCl_3_): δ = 7.81 (d, J= 7.2 Hz, 2H), 7.66 (s, 1H), 7.42-7.36 (m, 5H), 7.34-7.29 (m, 3H), 5.58 (s, 2H). ^13^C NMR (101 MHz, CDCl_3_): δ = 148.33, 134.75, 130.60, 129.26, 128.89, 128.26, 125.78, 119.55, 54.33. HRMS (ESI) calcd for C_15_H_13_N_3_ [M+H]^+^: 236.1109, found: 236.1186.





1,4-diphenyl-1*H*-1,2,3-triazole **(3b)**: White solid; Yield 85%; IR (ν_max_ cm^−1^): 3120, 3056, 2958, 2919, 2850, 1735, 1597, 1499; 1449, 1370, 1257, 1223 (N-N=N-); 1090, 1021, 917, 862, 798, 764, 685, 567. ^1^H NMR (400 MHz, CDCl_3_): δ = 8.20 (s, 1H), 7.92 (d, J= 7.0 Hz, 2H), 7.80 (d, J= 7.3 Hz, 2H), 7.56 (dd, J= 10.5, 5.0 Hz, 2H), 7.47 (dd, J= 10.0, 4.9 Hz, 2H), 7.40-7.35 (m, 2H). ^13^C NMR (101 MHz, CDCl_3_): δ = 148.41, 137.06, 130.21, 129.78, 128.92, 128.78, 125.84, 120.53, 117.58. HRMS (ESI) calcd for C_14_H_11_N_3_ [M+H]^+^: 222.0953, found: 222.1033.





1-Benzyl-4-(4-methoxy-phenyl)-1*H*-1,2,3-triazole **(3c)**: Off white solid; Yield 61%; IR (ν_max_

cm^−1^): 3133, 3037, 2964, 2958, 2851, 2835, 1613, 1579, 1557, 1453 (CH_2_), 1349, 1261 (N-N=N-), 1248, 1217, 1170 (C-N), 1070, 1026, 972, 833 (=C-H oop, triazole ring), 795, 717, 578. ^1^H NMR (400 MHz, CDCl_3_): δ = 7.71 (d, J= 8.9 Hz, 2H), 7.56 (s, 1H), 7.37 (d, J= 7.3 Hz, 3H), 7.31-7.28 (m, 2H), 6.92 (d, J= 8.9 Hz, 2H), 5.56 (s, 2H), 3.83 (s, 3H). ^13^C NMR (101 MHz, CDCl_3_): δ = 159.56, 148.08, 134.74, 129.12, 128.73, 128.03, 126.98, 123.24, 118.65, 114.18, 56.29, 54.18. HRMS (ESI) calcd for C_16_H_15_N_3_O [M+H]^+^: 266.1215, found: 266.1295.





1-Benzyl-4-*m*-tolyl-1*H*-1,2,3-triazole **(3d)**: Off white solid; Yield 88%; IR (ν_max_ cm^−1^): 3143, 2924, 2850, 1493, 1448 (CH_2_), 1421, 1346, 1218 (N-N=N-), 1176 (C-N), 1065, 1045, 976, 913, 843 (=C-H oop, triazole ring), 778, 716, 582, 482. ^1^H NMR (400 MHz, CDCl_3_): δ = 7.64 (d, J= 4.7 Hz, 2H), 7.56 (d, J= 7.7 Hz, 1H), 7.42-7.33 (m, 3H), 7.32-7.22 (m, 3H), 7.11 (d, J= 7.6 Hz, 1H), 5.55 (s, 2H), 2.36 (s, 3H). ^13^C NMR (101 MHz, CDCl_3_): δ = 148.32, 138.47, 134.67, 130.34, 129.13, 128.92, 128.67, 128.03, 126.34, 122.76, 119.43, 54.20, 21.38. HRMS (ESI) calcd for C_16_H_15_N_3_ [M+H]^+^: 250.1266, found: 250.1349.





4-(4-methoxyphenyl)-1-phenyl-1*H*-1,2,3-triazole **(3e)**: Pale yellow solid; Yield 59%; IR (ν_max_ cm^−1^): 3125, 3106, 3056, 2923, 2845, 1612, 1558, 1489; 1306, 1228 (N-N=N-); 1174, 1105, 1031, 912, 813, 759, 685, 606, 537. ^1^H NMR (400 MHz, CDCl_3_): δ = 8.10 (s, 1H), 7.83 (d, J= 8.8 Hz, 2H), 7.78 (d, J= 7.5 Hz, 2H), 7.54 (t, J= 7.8 Hz, 2H), 7.44 (t, J= 7.4 Hz, 1H), 6.99 (d, J= 8.8 Hz, 2H), 3.85 (s, 3H). ^13^C NMR (101 MHz, CDCl_3_): δ = 158.77, 147.99, 136.09, 128.73, 127.66, 126.14, 121.88, 119.46, 115.74, 113.29, 54.32. HRMS (ESI) calcd for C_15_H_13_N_3_O [M+H]^+^: 252.1059, found: 252.1134.





1-phenyl-4-(*m*-tolyl)-1*H*-1,2,3-triazole **(3f)**: Off white solid; Yield 84%; IR (ν_max_ cm^−1^): 3124, 2955, 2920, 2850, 1735, 1592, 1494, 1459; 1395, 1257, 1223 (N-N=N-); 1080, 1031, 902, 789, 754, 685, 661, 562. ^1^H NMR (400 MHz, CDCl_3_): δ = 8.19 (s, 1H), 7.80 (d, J= 8.3 Hz, 1H), 7.69 (d, J= 7.7 Hz, 3H), 7.60-7.53 (m, 2H), 7.50-7.43 (m, 1H), 7.35 (t, J= 7.6 Hz, 1H), 7.19 (d, J= 7.6 Hz, 1H), 2.43 (s, 3H). ^13^C NMR (101 MHz, CDCl_3_): δ = 147.50, 137.61, 129.05, 128.75, 128.18, 127.79, 127.72, 125.51, 121.91, 119.50, 116.52, 20.44. HRMS (ESI) calcd for C_15_H_13_N_3_ [M+H]^+^: 236.1109, found: 236.1184.





1-benzyl-4-(4-(trifluoromethyl)phenyl)-1*H*-1,2,3-triazole **(3g)**: Off white solid; Yield 64%; IR (ν_max_ cm^−1^): 3135, 3089, 3042, 2978, 2941, 1617, 1577, 1499, 1449 (CH_2_), 1431, 1321, 1229 (N-N=N-), 1159, 1105 (C-N), 1060, 1012, 971, 833 (=C-H oop, triazole ring), 779, 725, 690, 596. ^1^H NMR (600 MHz, CDCl_3_): δ = 7.91 (d, J= 8.1 Hz, 2H), 7.73 (s, 1H), 7.65 (d, J= 8.2 Hz, 2H), 7.49-7.36 (m, 3H), 7.33 (d, J= 6.4 Hz, 2H), 5.60 (s, 2H). ^13^C NMR (151 MHz, CDCl_3_): δ = 146.85, 134.35, 130.11, 129.89, 128.96, 128.14, 128.00, 127.00, 125.76, 120.19, 54.38. ESI-MS calcd for C_16_H_12_F_3_N_3_ [M+H]^+^: 304.0983, found: 304.4400.





1-Benzyl-4-(4-fluoro-phenyl)-1*H*-1,2,3-triazole **(3h)**: Off white solid; Yield 53%; ^1^H NMR (600 MHz, CDCl_3_): δ = 7.77 (dd, J= 8.8, 5.4 Hz, 2H), 7.61 (s, 1H), 7.39 (t, J= 7.7 Hz, 3H), 7.31 (d, J= 6.4 Hz, 2H), 7.09 (t, J= 8.7 Hz, 2H), 5.57 (s, 2H). ^13^C NMR (151 MHz, CDCl_3_): δ = 163.26, 161.62, 147.17, 134.36, 128.97, 128.63, 127.87, 127.24, 127.18, 126.54, 118.96, 115.64, 115.49, 54.09.





1-phenyl-4-(4-(trifluoromethyl)phenyl)-1*H*-1,2,3-triazole **(3i)**: Off white; Yield 50%; IR (ν_max_ cm^−1^): 3111, 2958, 2923, 2845, 1735, 1622, 1592, 1508, 1469; 1430, 1321, 1262, 1223 (N-N=N-); 1159, 1100, 1011, 912, 843, 818, 754, 680, 596, 532. ^1^H NMR (400 MHz, CDCl_3_): δ = 8.27 (s, 1H), 8.03 (d, J= 8.1 Hz, 2H), 7.79 (d, J= 7.9 Hz, 2H), 7.72 (d, J= 8.2 Hz, 2H), 7.56 (t, J= 7.7 Hz, 2H), 7.48 (t, J= 7.4 Hz, 1H). HRMS (ESI) calcd for C_15_H_10_F_3_N_3_ [M+H]^+^: 290.0827, found: 290.1103.





1-Benzyl-4-(thiophen-2-yl)-1*H*-1,2,3-triazole **(3j)**: White solid; Yield 90%; IR (ν_max_ cm^−1^): 3135, 3081, 2919, 2854, 1651, 1582, 1489, 1454 (CH_2_), 1435, 1361, 1306, 1257 (N-N=N-), 1208, 1178 (C-N), 1045, 926, 833 (=C-H oop, triazole ring), 716, 692, 582. ^1^H NMR (400 MHz, CDCl_3_): δ = 7.77 (s, 1H), 7.61-7.56 (m, 3H), 7.55-7.45 (m, 4H), 7.25 (dd, J= 5.1, 3.6 Hz, 1H), 5.76 (s, 2H). ^13^C NMR (101 MHz, CDCl_3_): δ = 142.28, 133.44, 131.82, 128.16, 127.84, 127.07, 126.56, 124.03, 123.13, 117.94, 53.25. HRMS (ESI) calcd for C_13_H_11_N_3_S [M+H]^+^: 242.0674, found: 242.0817.





1-phenyl-4-(thiophen-2-yl)-1*H*-1,2,3-triazole **(3k)**: Brown soilid; Yield 87%; IR (ν_max_ cm^−1^): 3125, 2958, 2923, 2850, 1592, 1499, 1459; 1425, 1370, 1282, 1257 (N-N=N-); 1228, 1154, 1085, 1036, 986, 926, 843, 798, 754, 680, 518. ^1^H NMR (400 MHz, CDCl_3_): δ = 8.09 (s, 1H), 7.78-7.75 (m, 3H), 7.55-7.45 (m, 3H), 7.33 (dd, J= 5.1, 1.1 Hz, 1H), 7.10 (dd, J= 5.1, 3.6 Hz, 1H). ^13^C NMR (101 MHz, CDCl_3_): δ = 143.48, 136.84, 132.43, 129.79, 128.88, 127.72, 125.38, 124.53, 120.53, 117.06. ESI-MS calcd for C_14_H_12_N_4_ [M+H]^+^: 228.0517, found: 228.3400





3-(1-benzyl-1*H*-1,2,3-triazol-4-yl)pyridine **(3l)**: Off white solid; Yield 85%; IR (ν_max_ cm^−1^): 3140, 3111, 3086, 3027, 2943, 2850, 1602, 1574, 1446 (CH_2_), 1417, 1334, 1218 (N-N=N-), 1178, 1080 (C-N), 1045, 971, 848 (=C-H oop, triazole ring), 803, 700, 659, 582. ^1^H NMR (600 MHz, CDCl_3_): δ = 8.95 (s, 1H), 8.55 (s, 1H), 8.17 (d, J= 7.9 Hz, 1H), 7.75 (s, 1H), 7.43-7.31 (m, 6H), 5.59 (s, 2H). ^13^C NMR (151 MHz, CDCl_3_): δ = 149.55, 147.33, 145.52, 134.74, 133.42, 129.64, 129.35, 128.52, 127.15, 124.20, 120.27, 54.78. ESI-MS calcd for C_14_H_12_N_4_ [M+H]^+^: 237.1062, found: 237.3900





Methyl 1-benzyl-1*H*-1,2,3-triazole-4-carboxylate **(3m)**: Off white solid; Yield 92%; IR (ν_max_ cm^−1^): 3112, 3071, 3002, 2948, 2919, 2845, 1543, 1494, 1454 (CH_2_), 1433, 1366, 1336, 1227 (N-N=N-), 1139, 1097, 1020 (C-N), 950, 882, 811 (=C-H oop, triazole ring), 778, 710, 692, 582, 474. ^1^H NMR (600 MHz, CDCl_3_): δ = 7.96 (s, 1H), 7.41-7.38 (m, 3H), 7.28 (d, J= 7.4 Hz, 2H), 5.56 (s, 2H), 3.91 (s, 3H). ^13^C NMR (151 MHz, CDCl_3_): δ = 161.18, 140.40, 133.70, 129.44, 128.39, 127.46, 54.59, 52.30. HRMS (ESI) calcd for C_11_H_11_N_3_O_2_ [M+H]^+^: 218.0851, found: 218.0934.





Methyl 1-phenyl-1*H*-1,2,3-triazole-4-carboxylate **(3n)**: off white solid; Yield 90%; IR (ν_max_ cm^−1^): 3135, 3076, 2997, 2938, 2845, 1597, 1543, 1508; 1439, 1351, 1262 (N-N=N-); 1203, 1149, 1036, 986, 907, 862, 808, 772, 750, 680, 631. ^1^H NMR (400 MHz, CDCl_3_): δ = 8.51 (s, 1H), 7.74 (d, J= 7.1 Hz, 2H), 7.57-7.52 (m, 2H), 7.48 (t, J= 7.4 Hz, 1H), 3.98 (s, 3H). ^13^C NMR (101 MHz, CDCl_3_): δ = 161.14, 140.63, 136.40, 130.06, 129.67, 125.67, 120.90, 52.49. HRMS (ESI) calcd for C_10_H_9_N_3_O_2_ [M+H]^+^: 204.0695, found: 204.0771.

1. **^1^H NMR and ^13^C NMR Spectra of 1,4-disubstituted 1,2,3-triazoles**

^1^H NMR and ^13^C NMR of 1-Benzyl-4-phenyl-1*H*-1,2,3-triazole (**3a**)


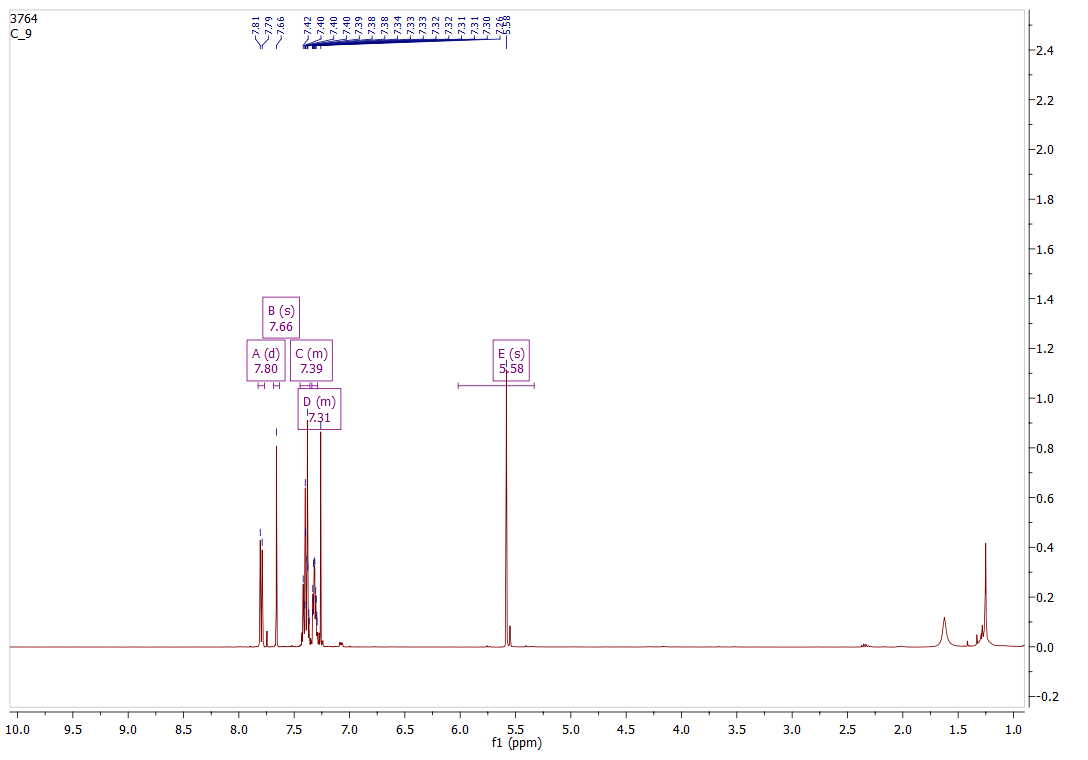


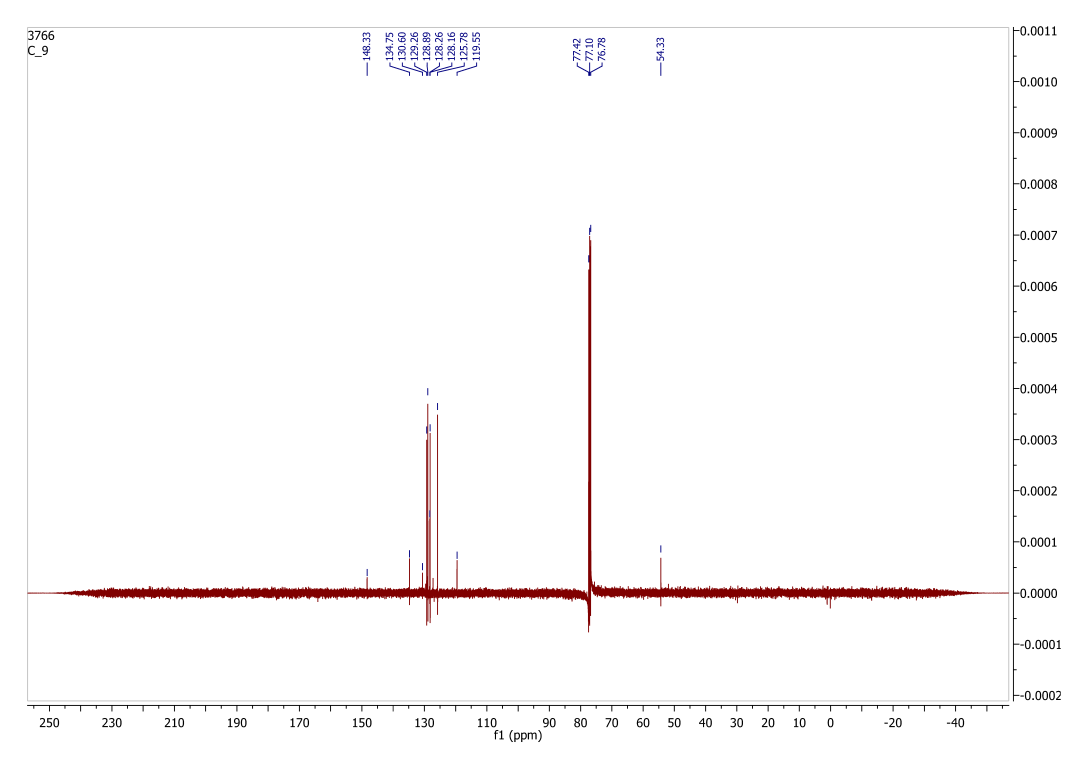


^1^H NMR and ^13^C NMR of 1,4-diphenyl-1*H*-1,2,3-triazole **(3b)**


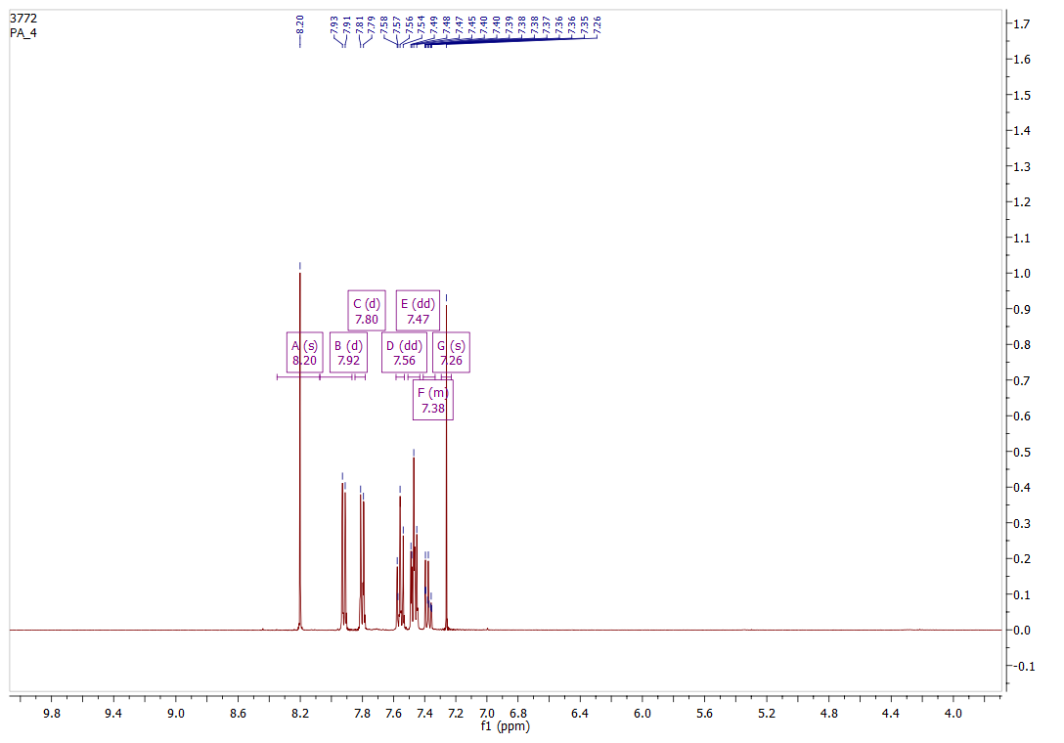


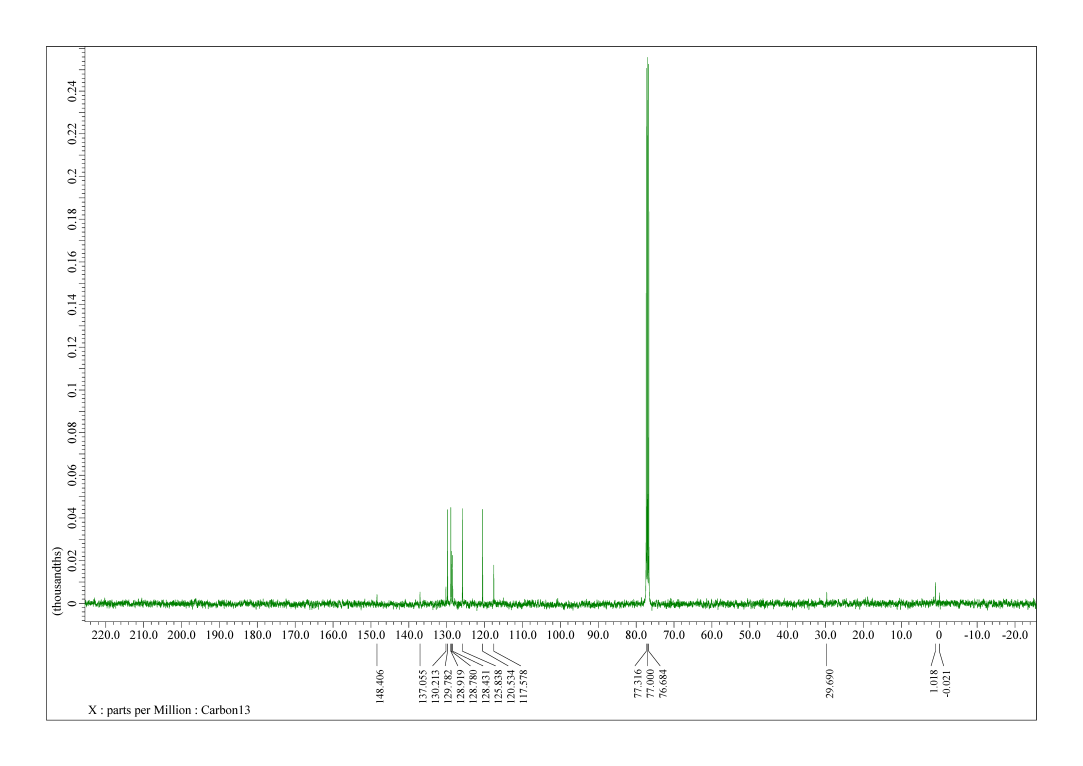


^1^H NMR and ^13^C NMR of 1-Benzyl-4-(4-methoxy-phenyl)-1*H*-1,2,3-triazole **(3c)**


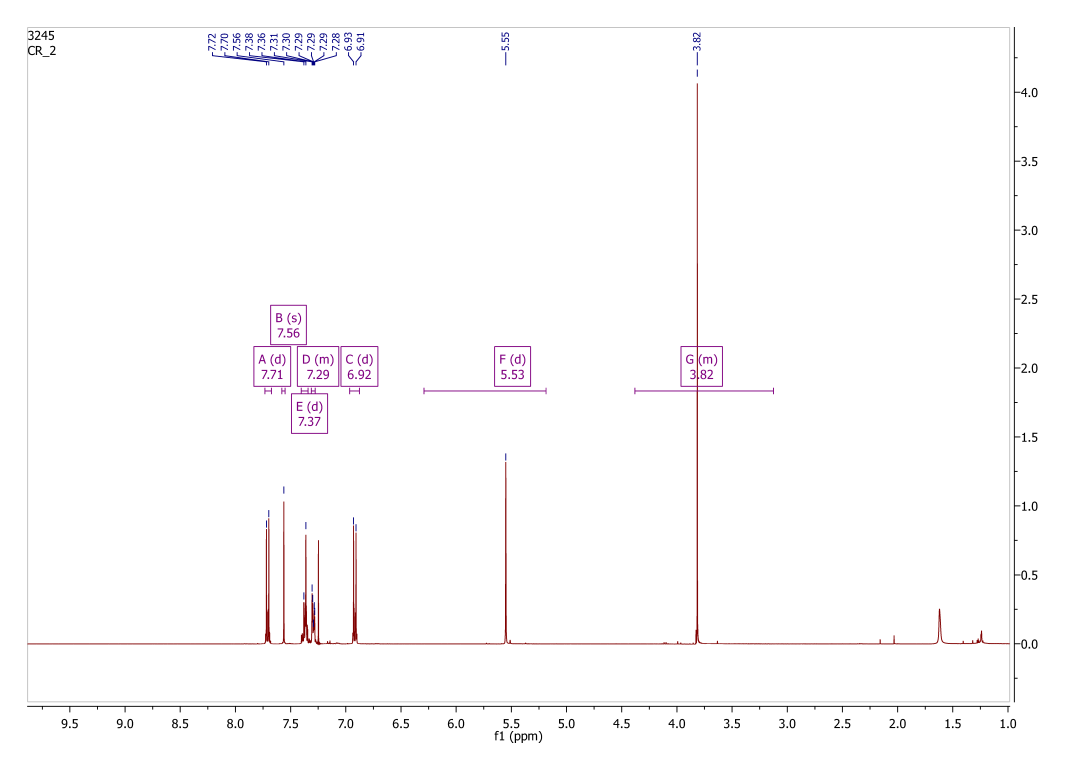


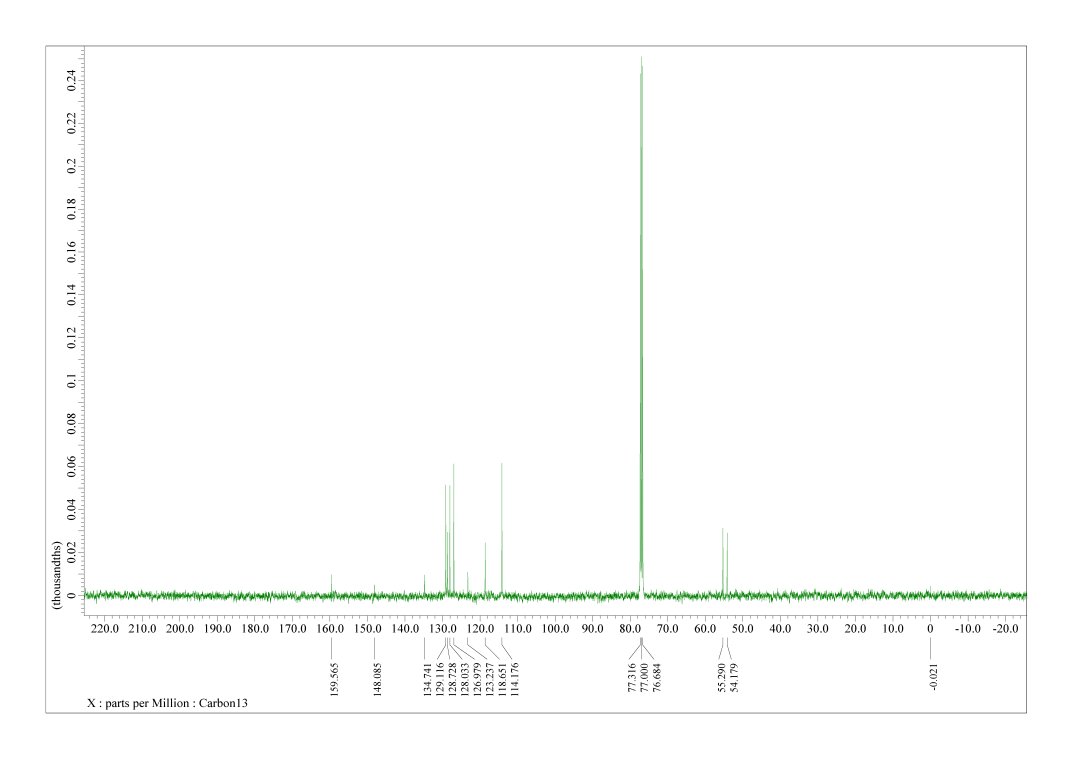


^1^H NMR and ^13^C NMR of 1-Benzyl-4-*m*-tolyl-1*H*-1,2,3-triazole **(3d)**


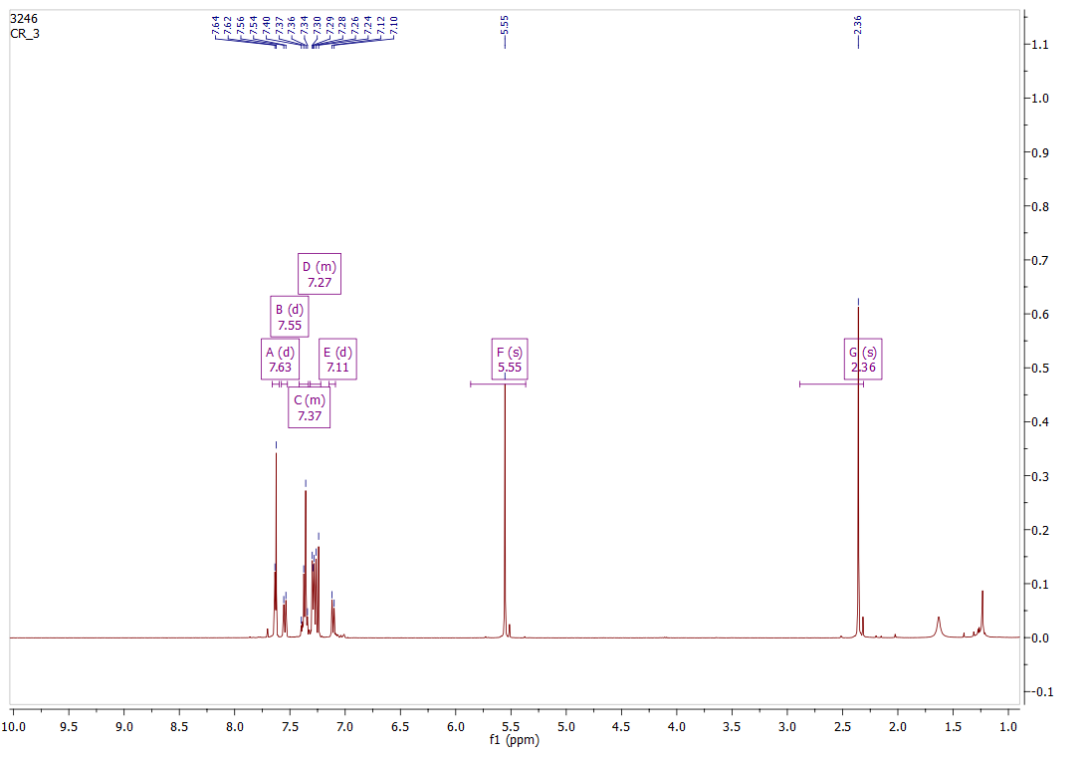


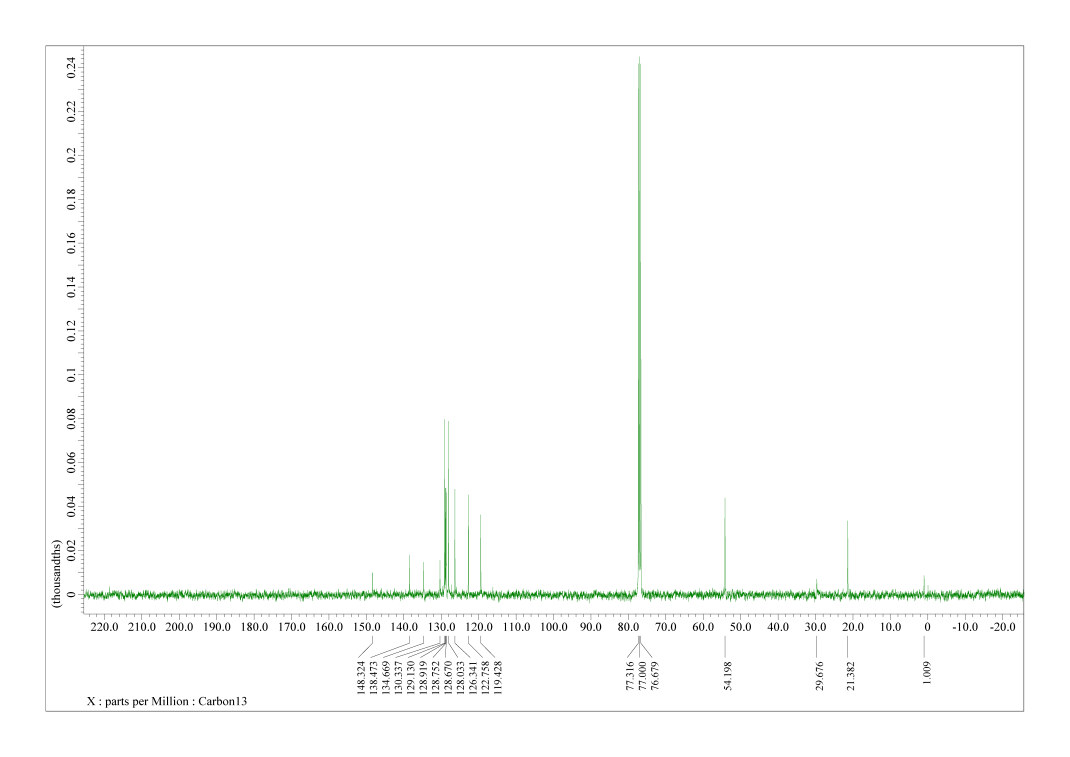


^1^H NMR and ^13^C NMR of 4-(4-methoxyphenyl)-1-phenyl-1*H*-1,2,3-triazole **(3e)**


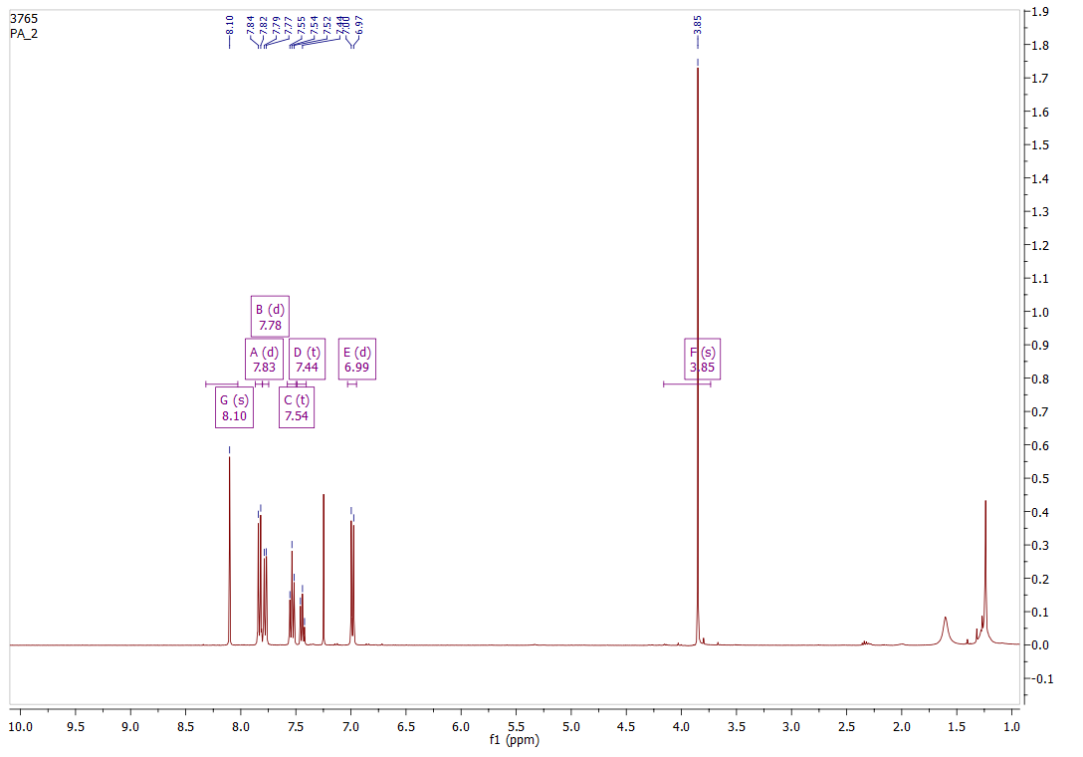


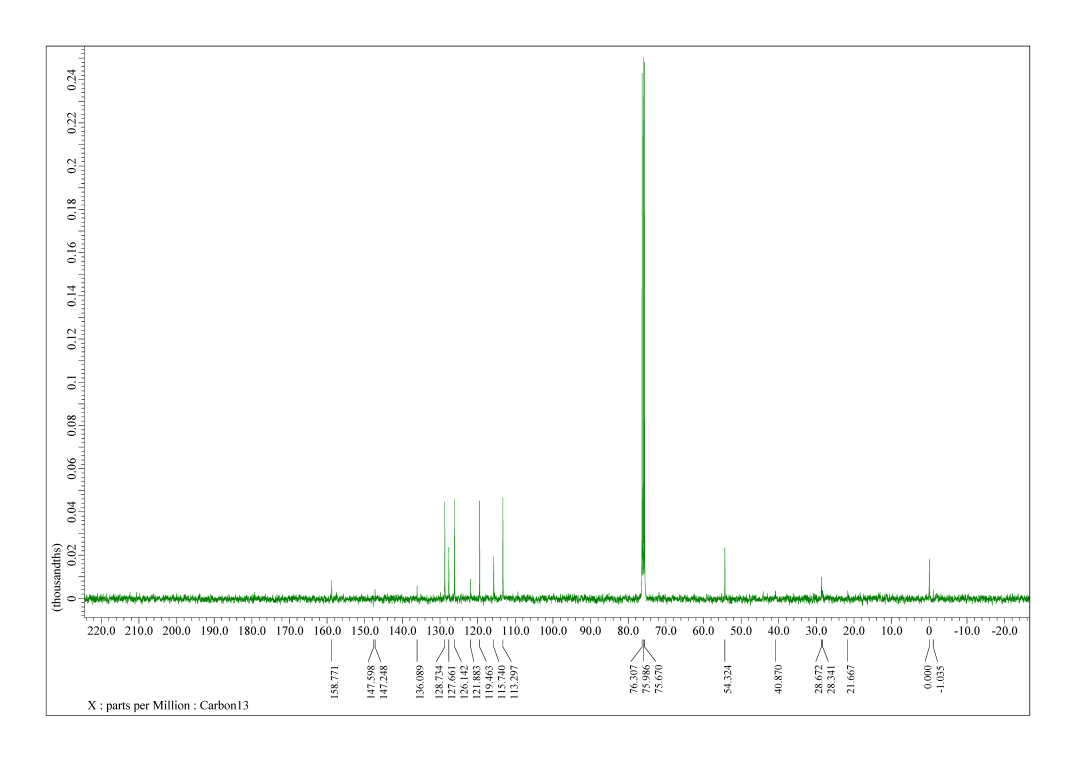


^1^H NMR and ^13^C NMR of 1-phenyl-4-(*m*-tolyl)-1*H*-1,2,3-triazole **(3f)**


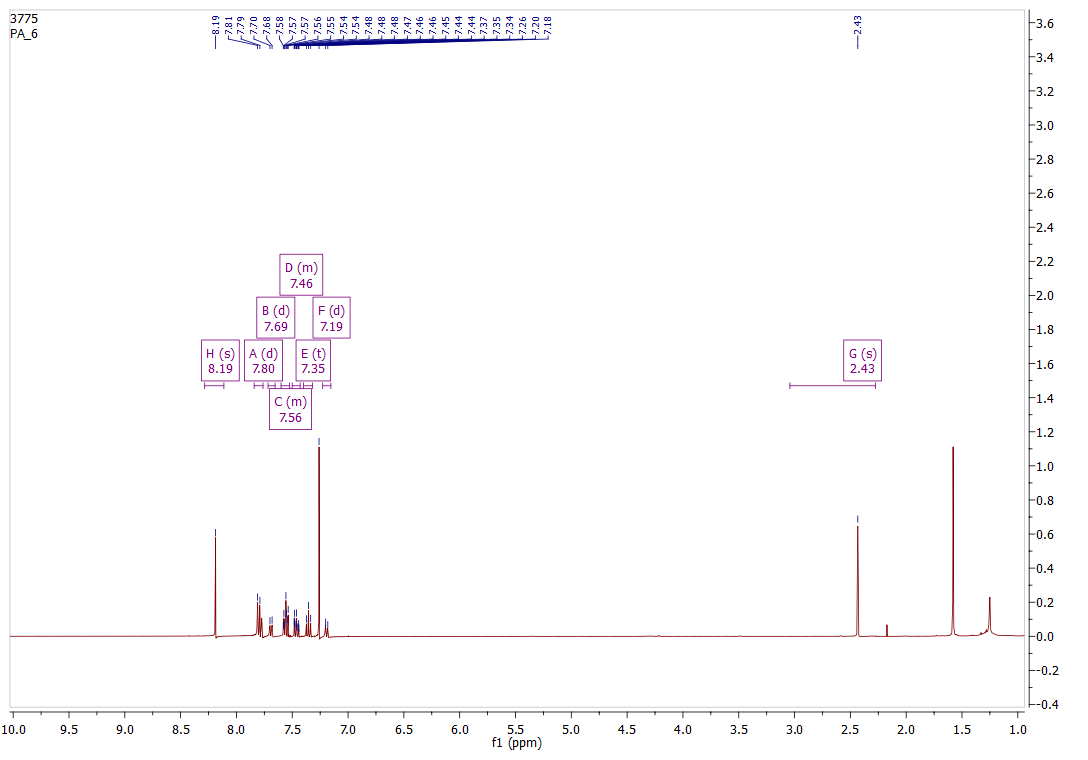


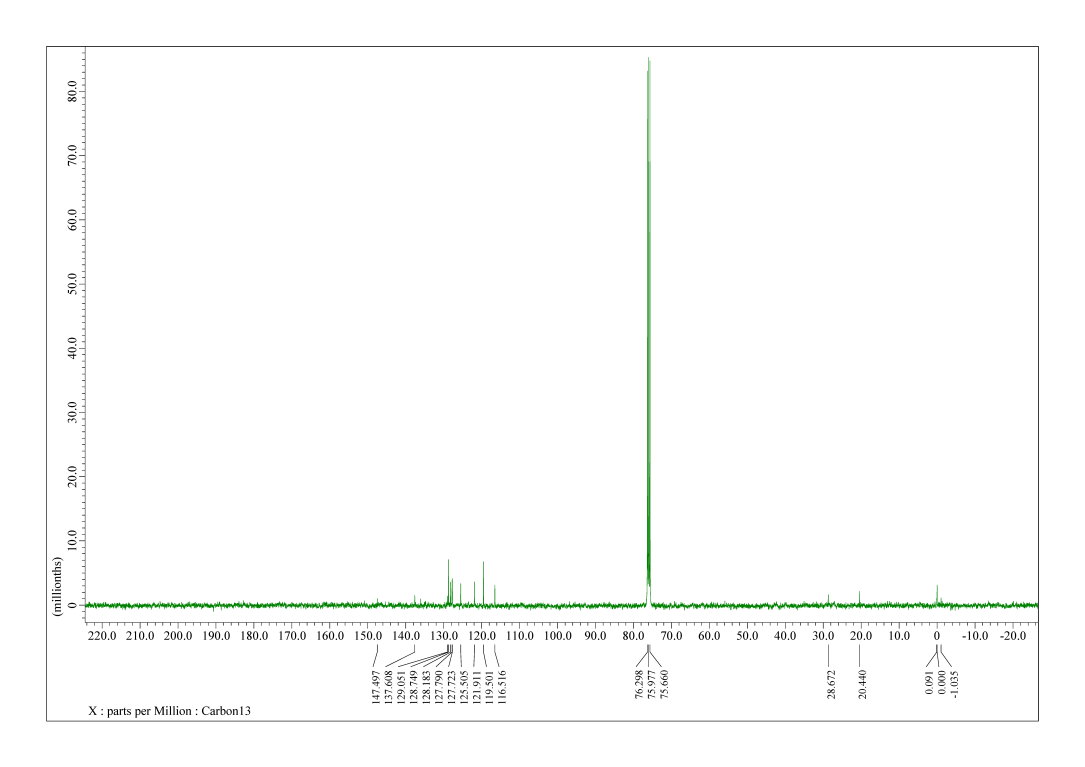


^1^H NMR and ^13^C NMR of 1-benzyl-4-(4-(trifluoromethyl)phenyl)-1*H*-1,2,3-triazole **(3g)**


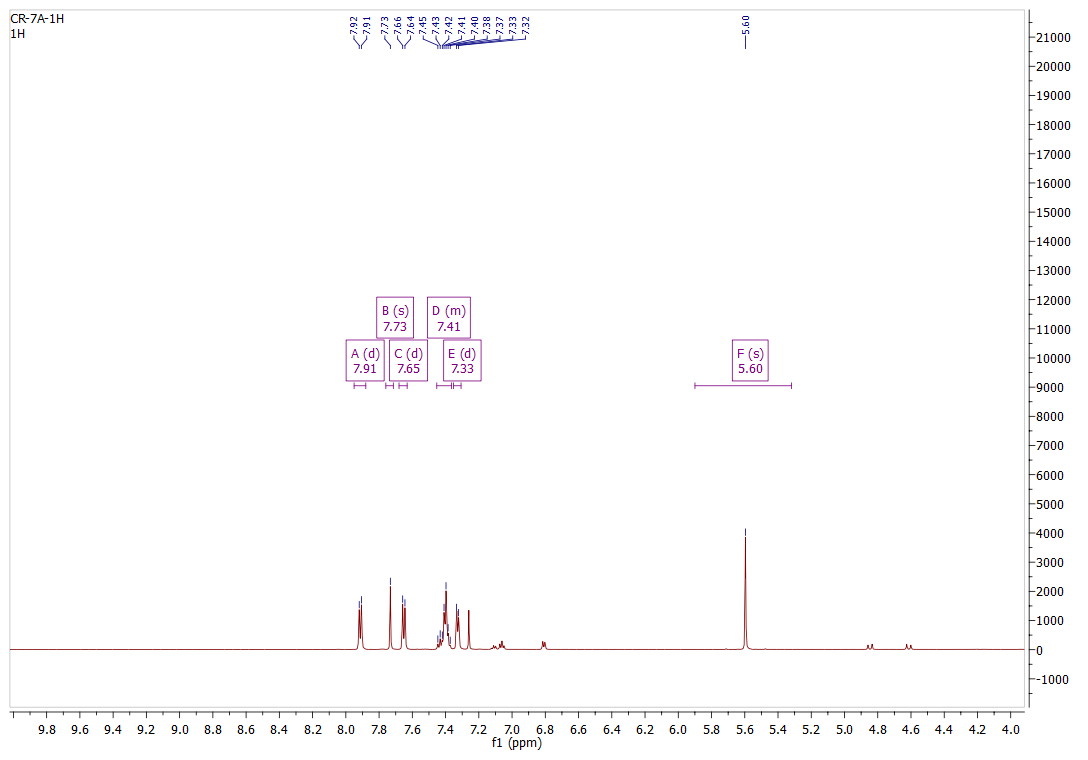


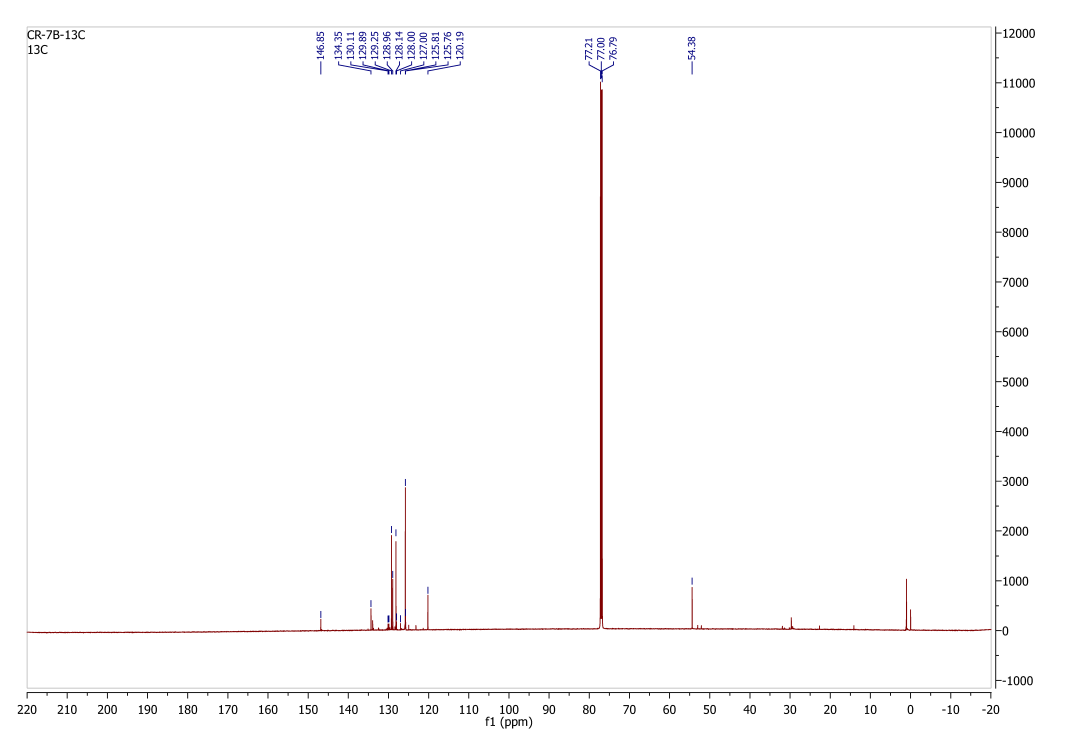


^1^H NMR and ^13^C NMR of 1-Benzyl-4-(4-fluoro-phenyl)-1*H*-1,2,3-triazole **(3h)**


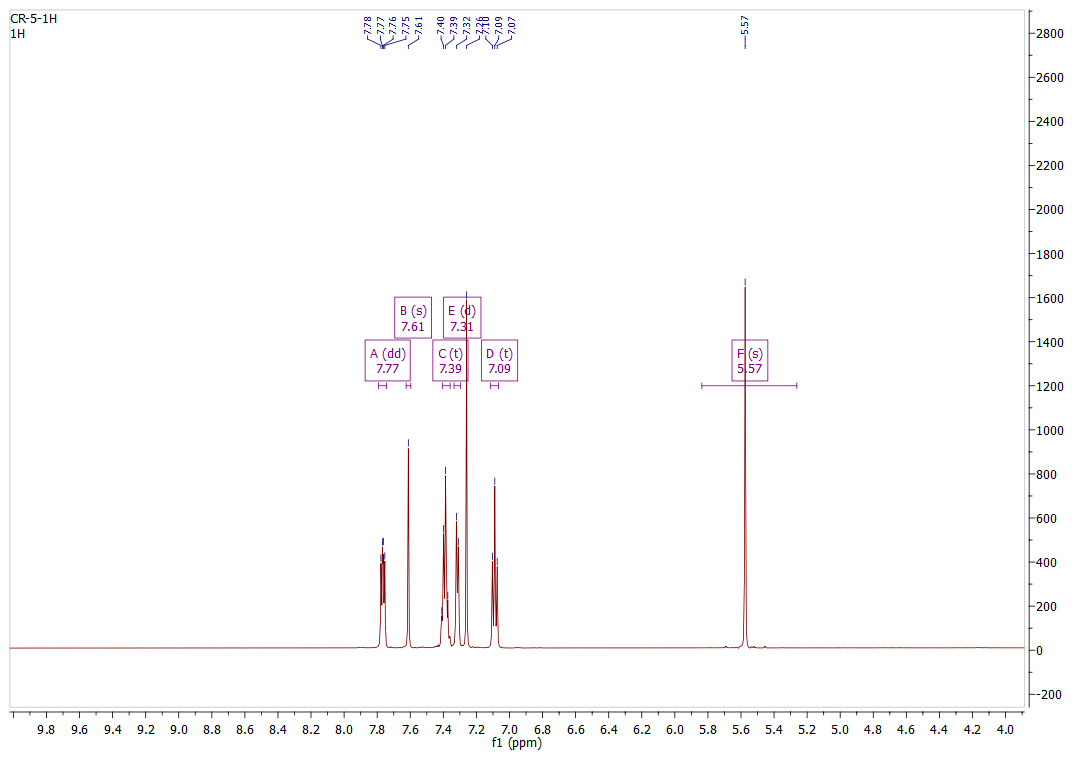


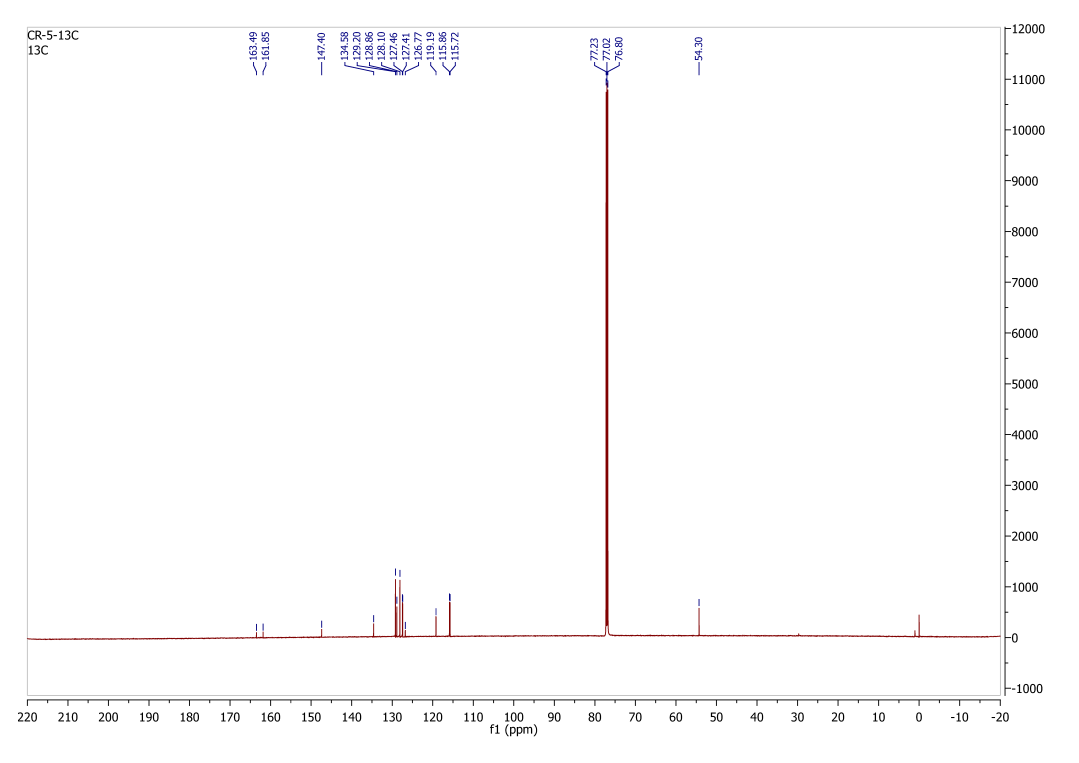


^1^H NMR and ^13^C NMR of 1-phenyl-4-(4-(trifluoromethyl)phenyl)-1*H*-1,2,3-triazole **(3i)**


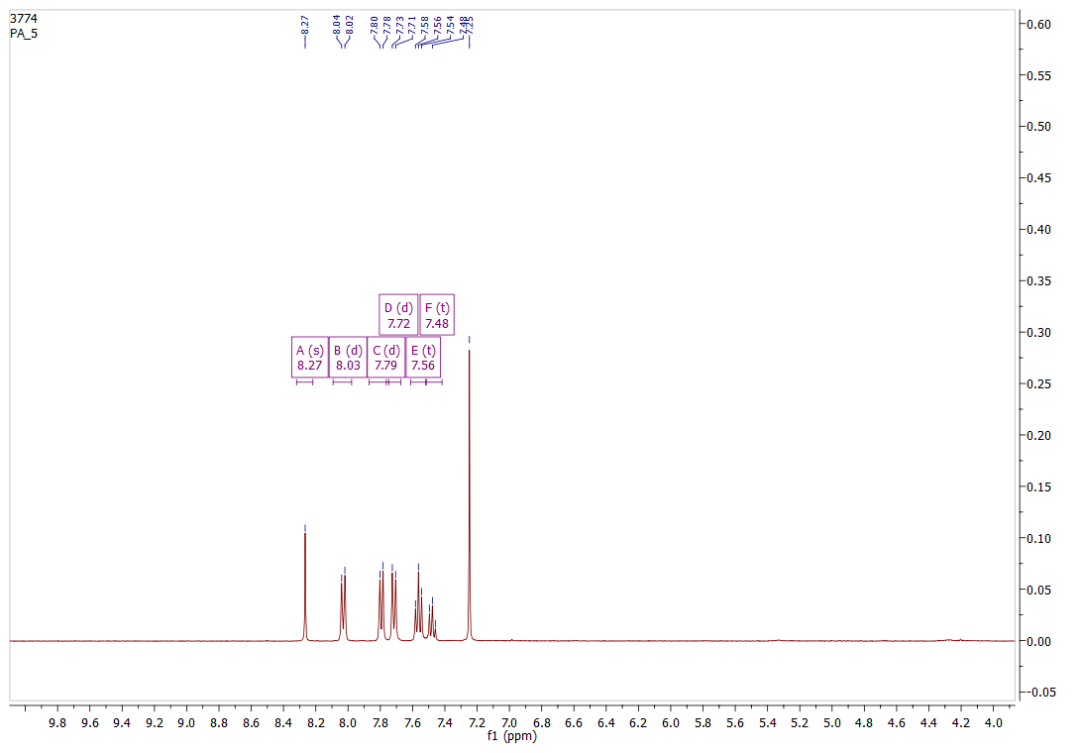


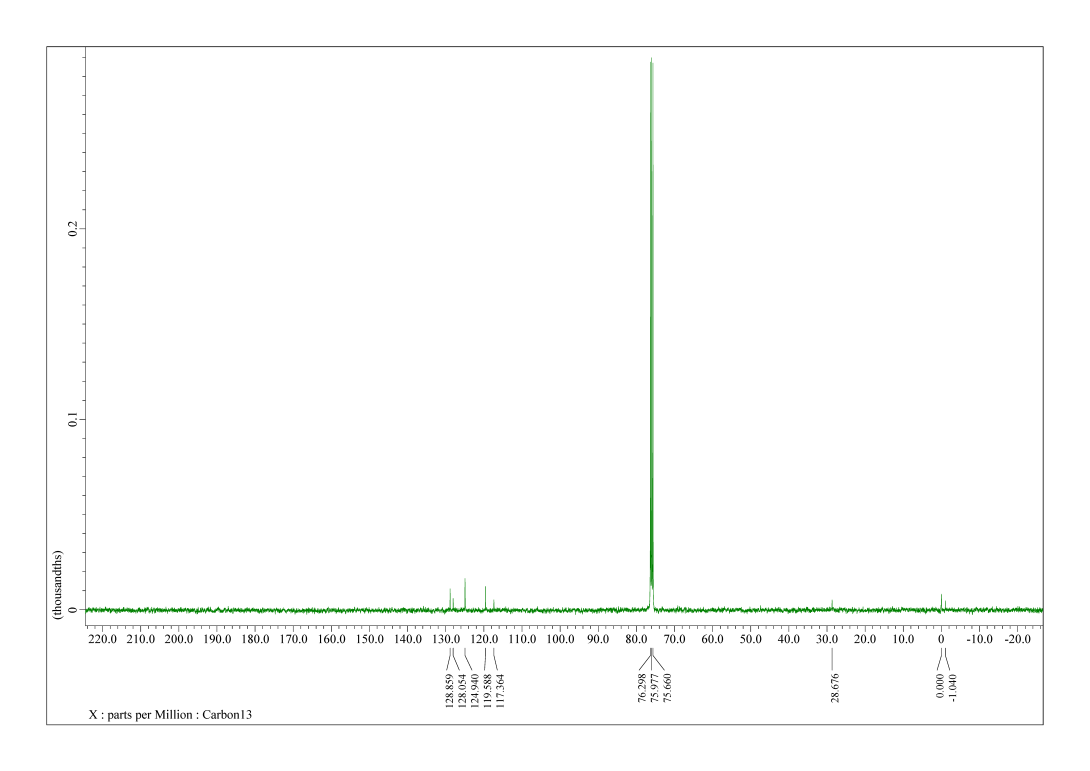


^1^H NMR and ^13^C NMR of 1-Benzyl-4-(thiophen-2-yl)-1*H*-1,2,3-triazole **(3j)**


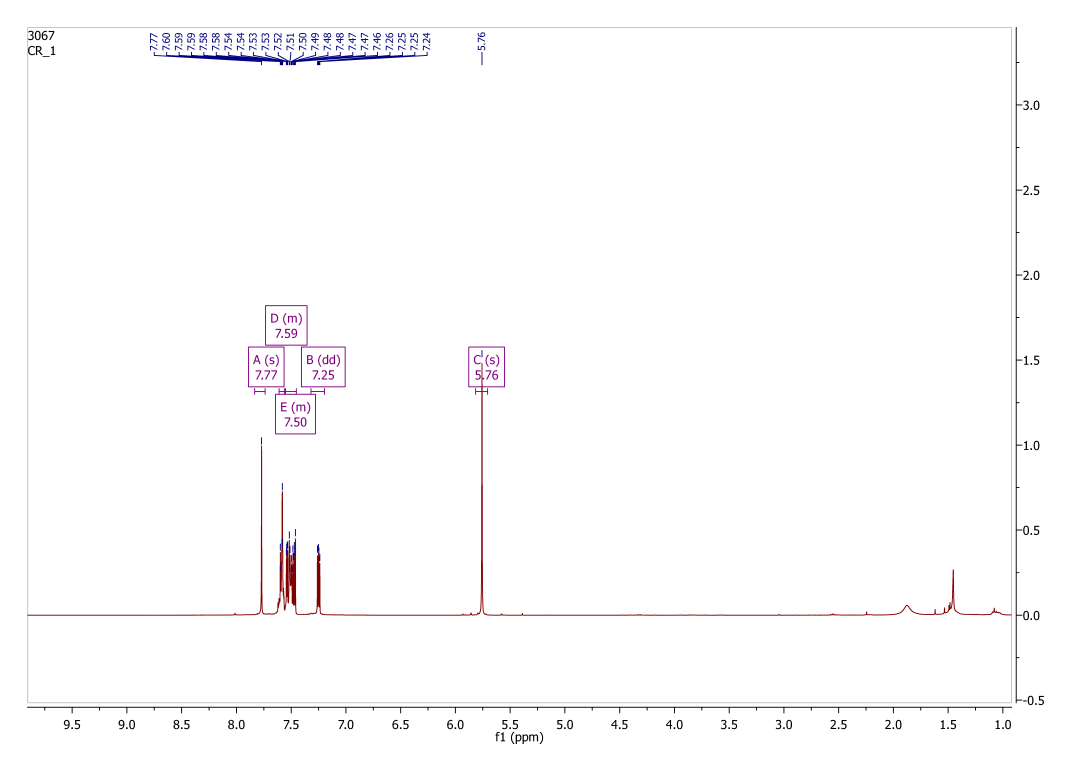


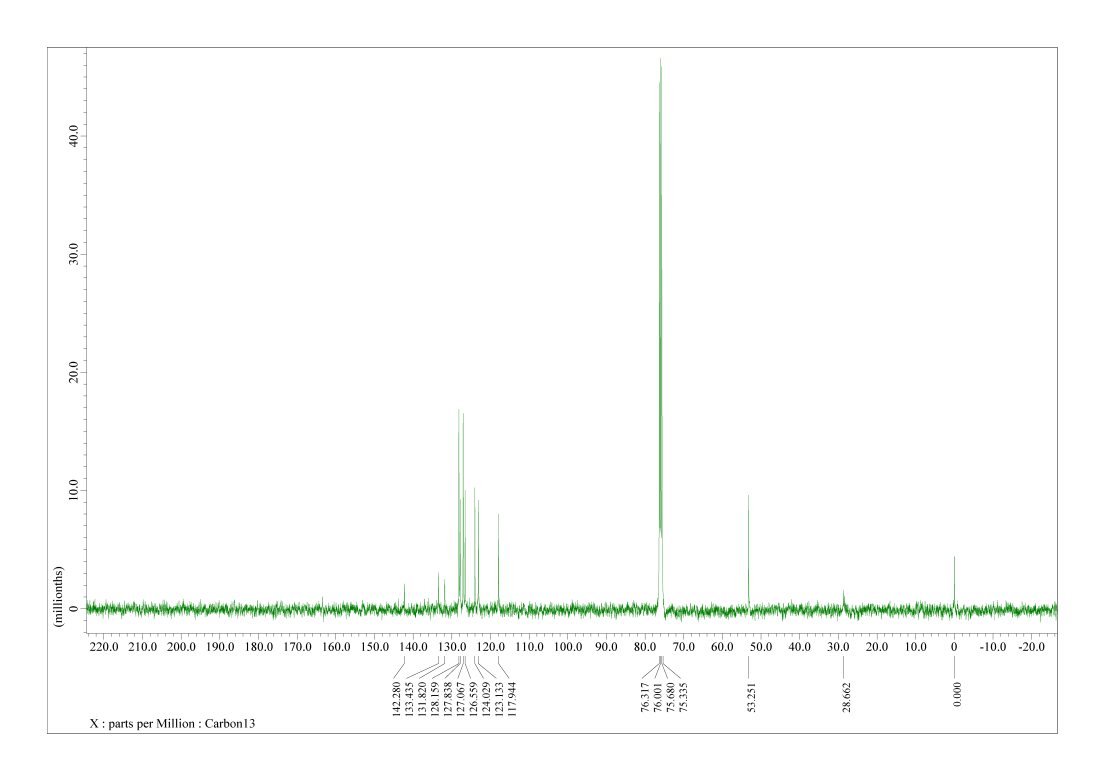


^1^H NMR and ^13^C NMR of 1-phenyl-4-(thiophen-2-yl)-1*H*-1,2,3-triazole **(3k)**


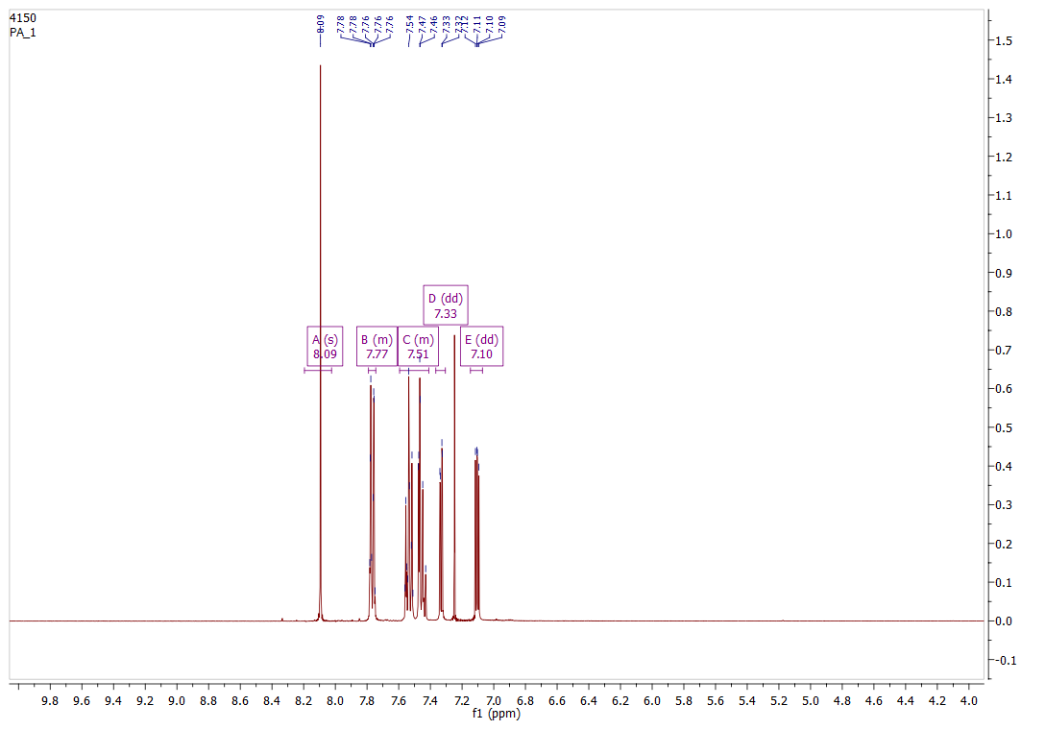


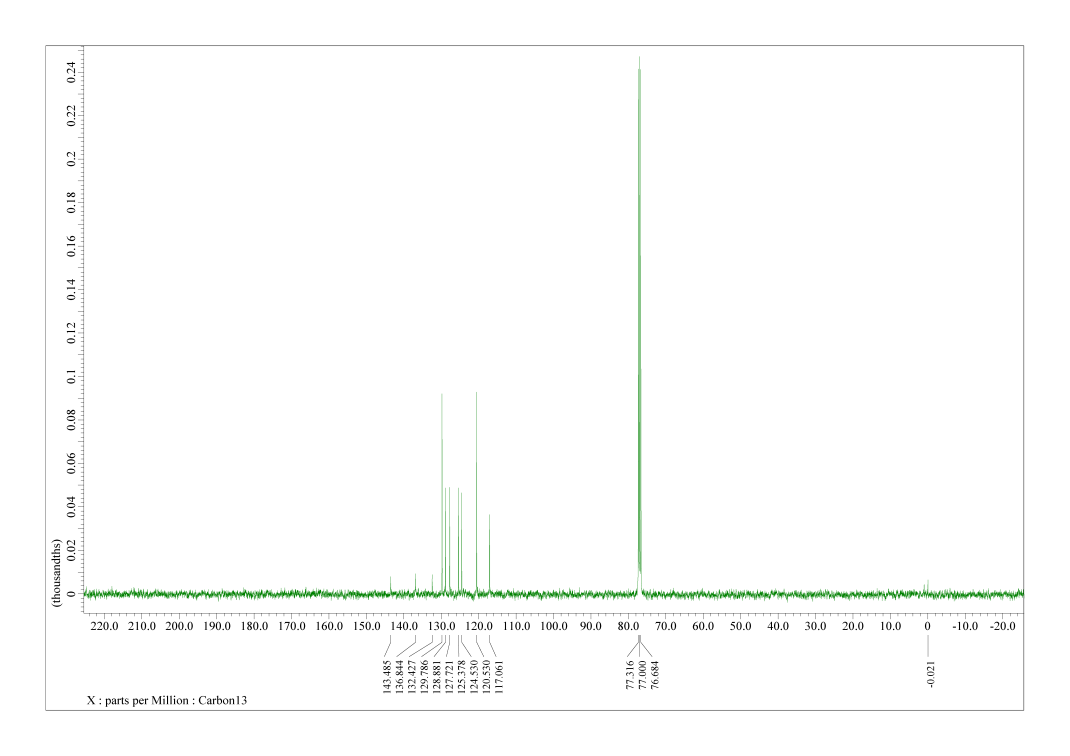


^1^H NMR and ^13^C NMR of 3-(1-benzyl-1*H*-1,2,3-triazol-4-yl)pyridine **(3l)**


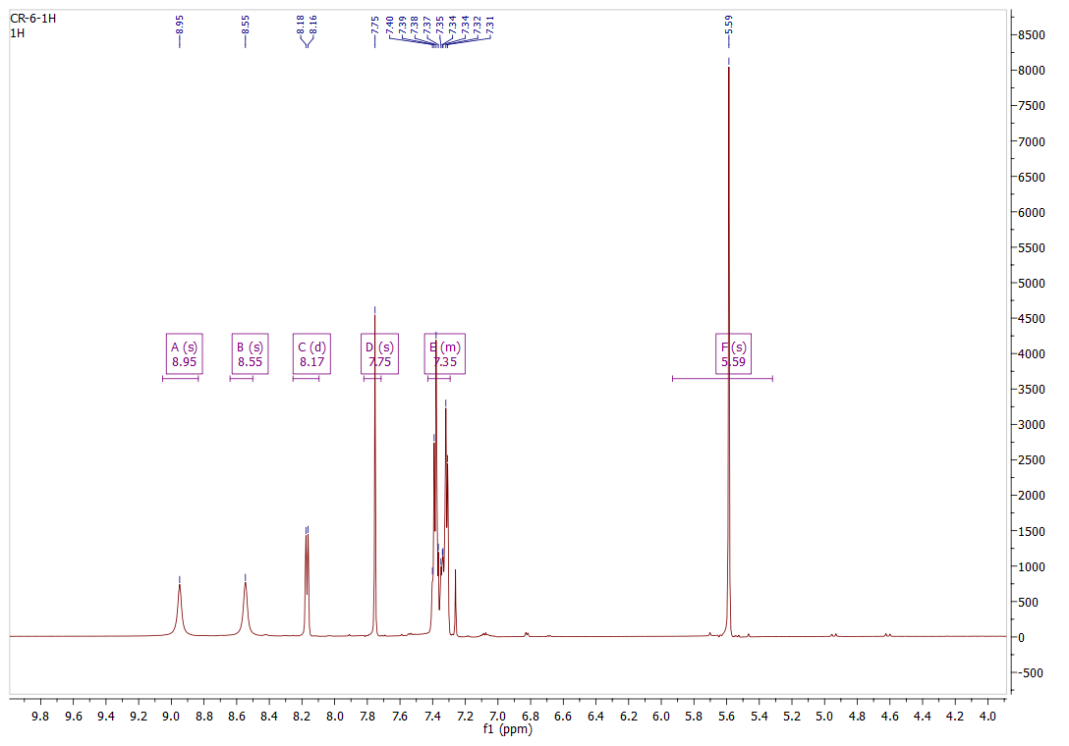


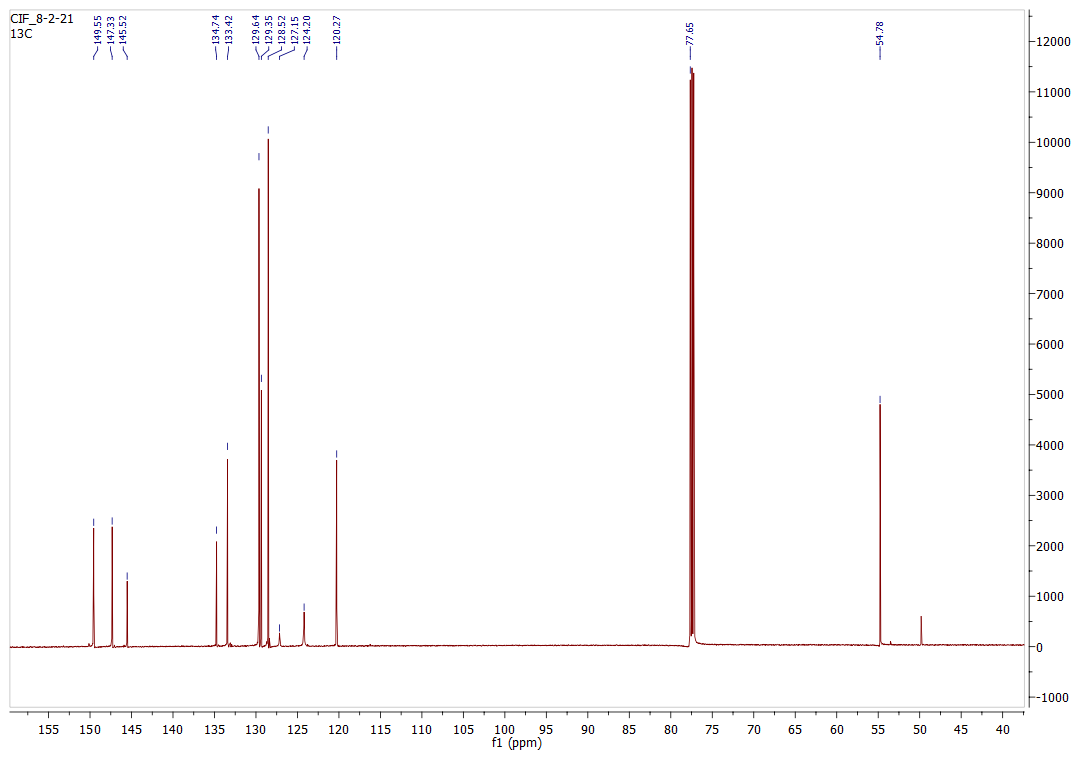


^1^H NMR and ^13^C NMR of Methyl 1-benzyl-1*H*-1,2,3-triazole-4-carboxylate **(3m)**


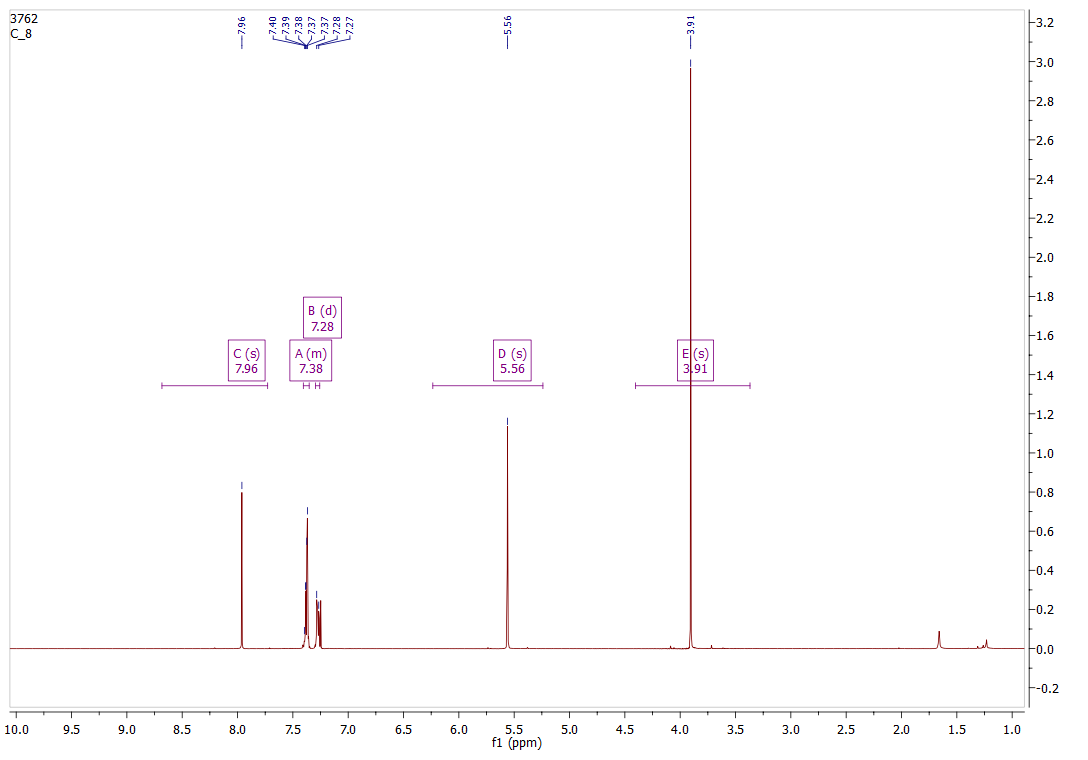


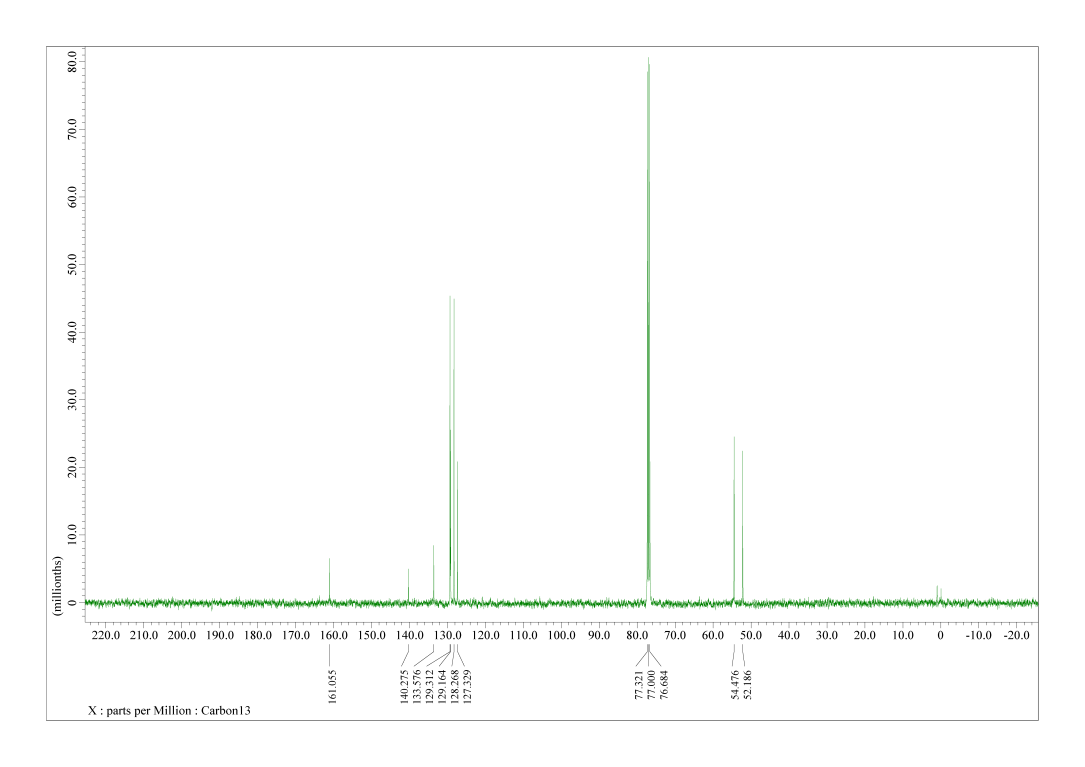


^1^H NMR and ^13^C NMR of Methyl 1-phenyl-1*H*-1,2,3-triazole-4-carboxylate **(3n)**


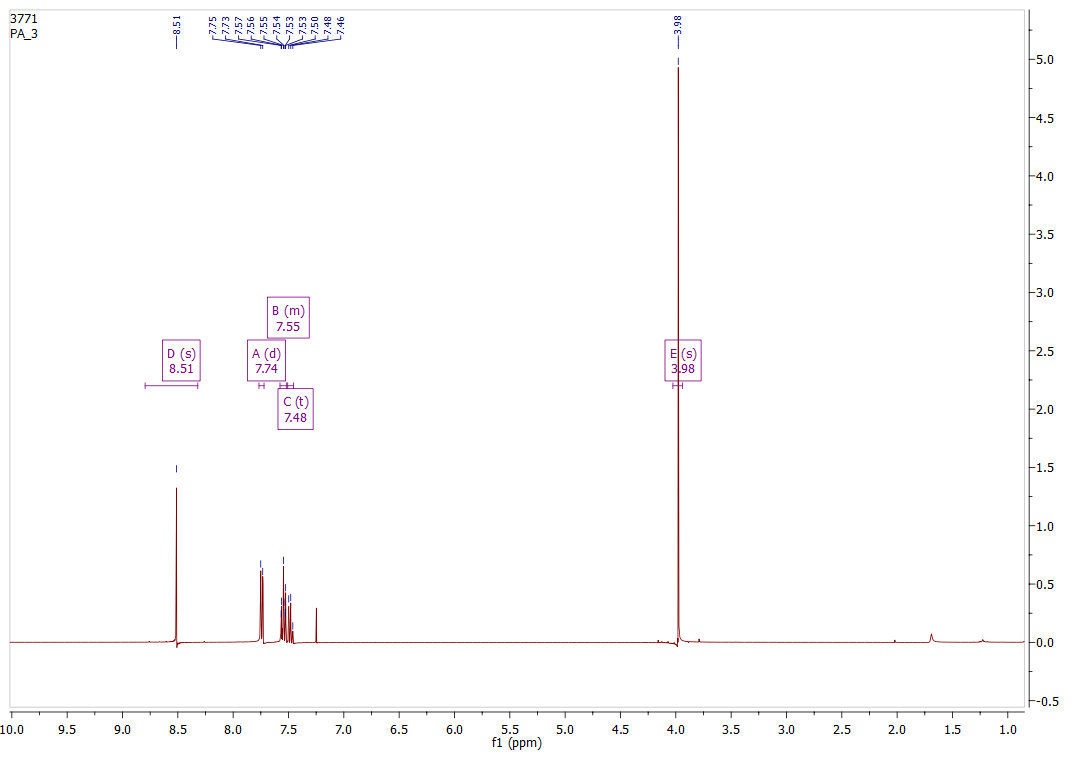


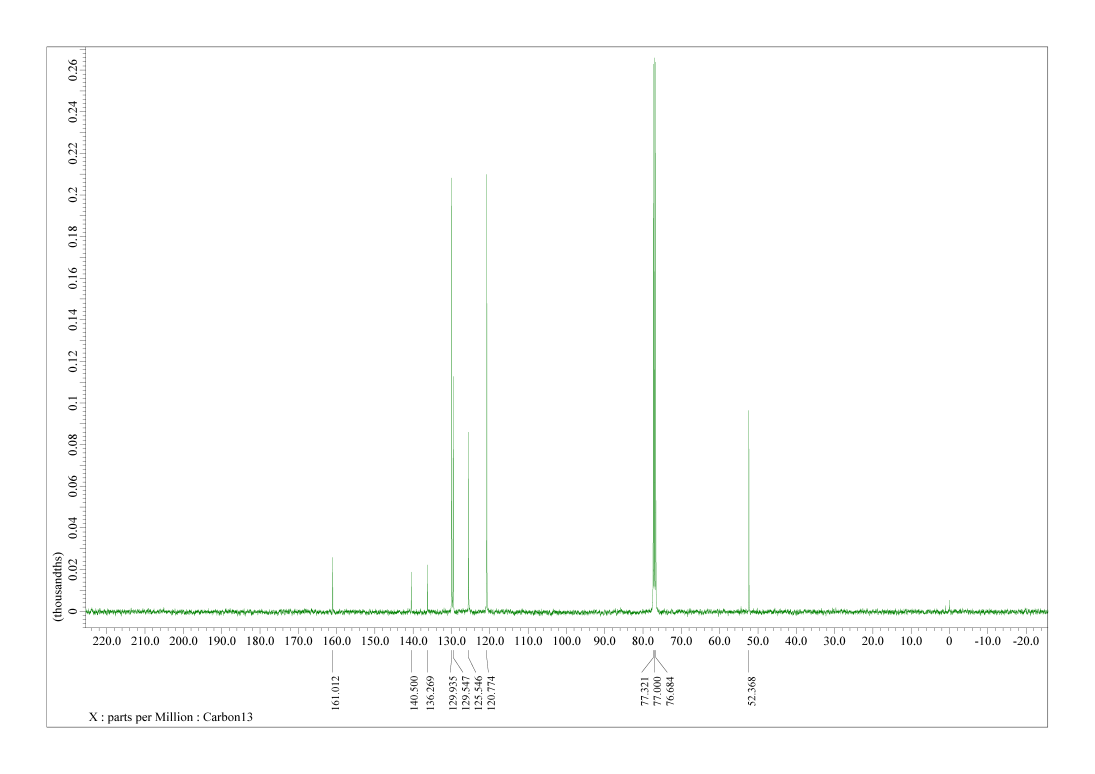


1. **FT-IR Spectra of 1,4-disubstituted 1,2,3-triazoles**

1-Benzyl-4-phenyl-1*H*-1,2,3-triazole (**3a**)


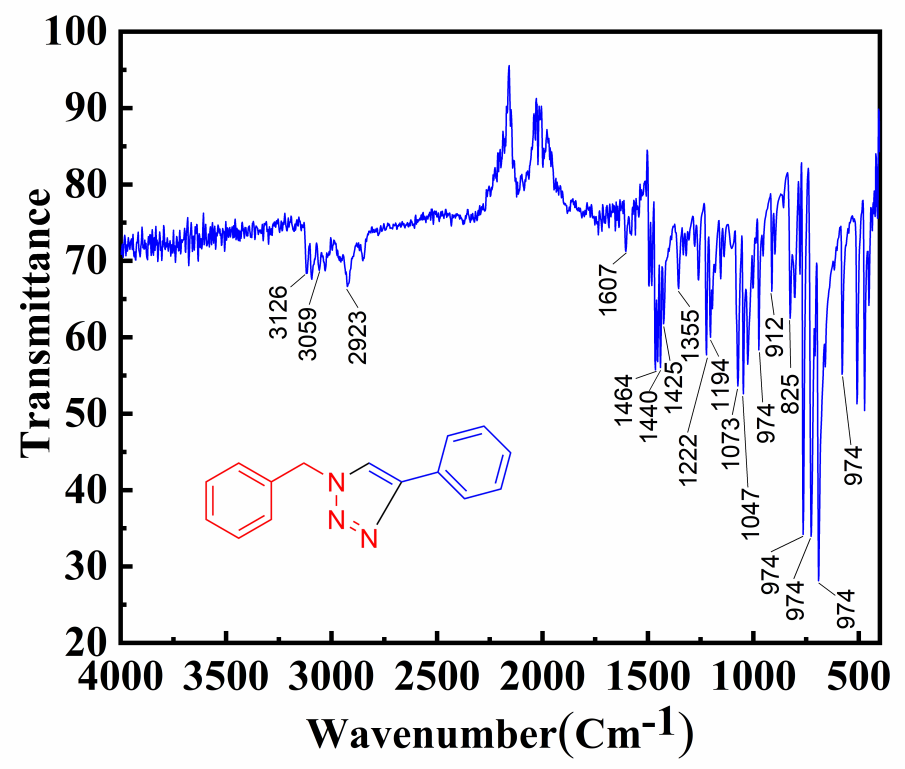


1,4-diphenyl-1*H*-1,2,3-triazole **(3b)**


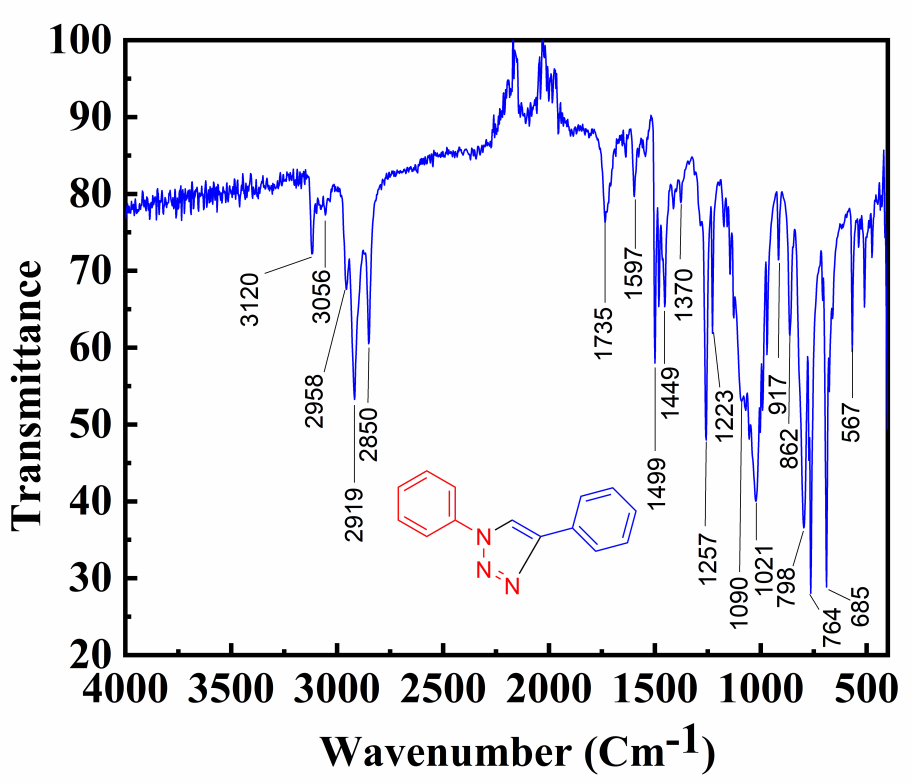


1-Benzyl-4-(4-methoxy-phenyl)-1*H*-1,2,3-triazole **(3c)**


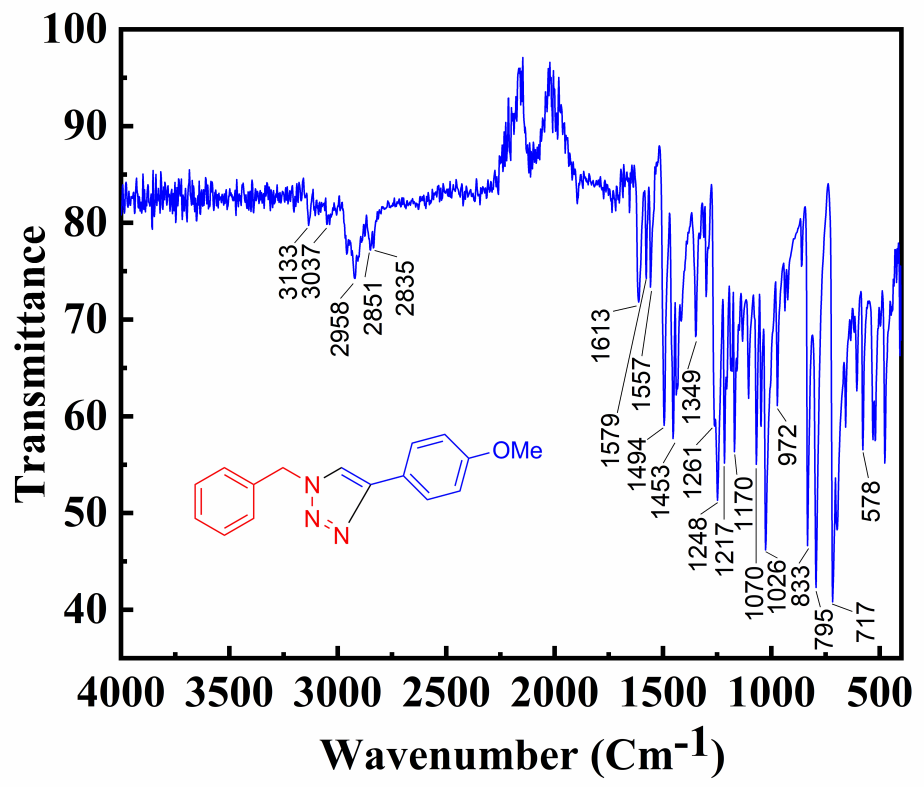


1-Benzyl-4-*m*-tolyl-1*H*-1,2,3-triazole **(3d)**


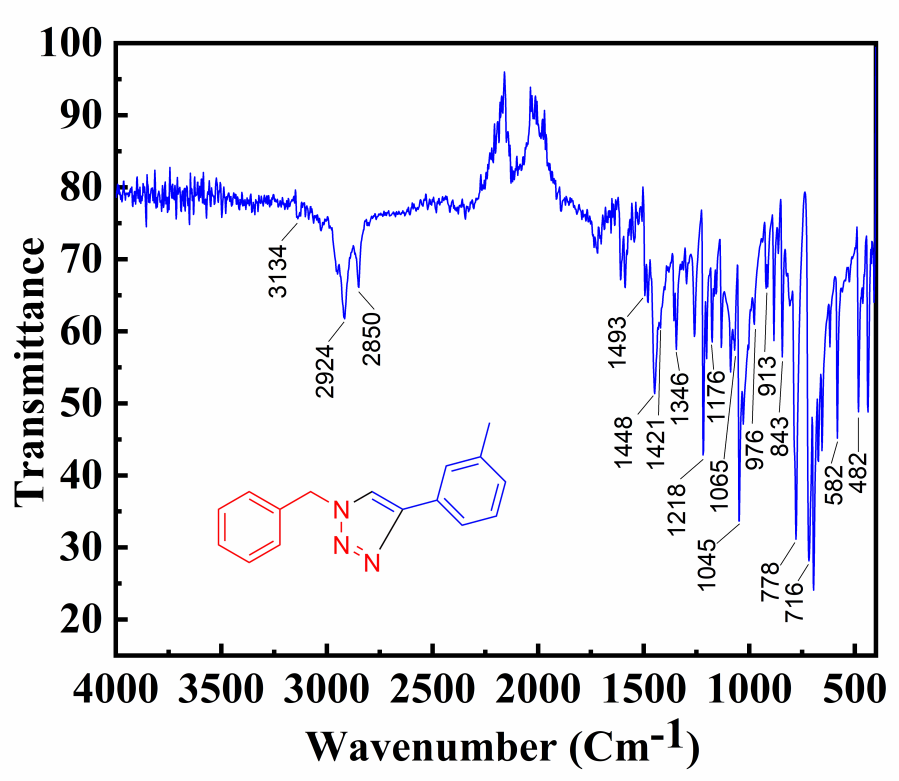


4-(4-methoxyphenyl)-1-phenyl-1*H*-1,2,3-triazole **(3e)**


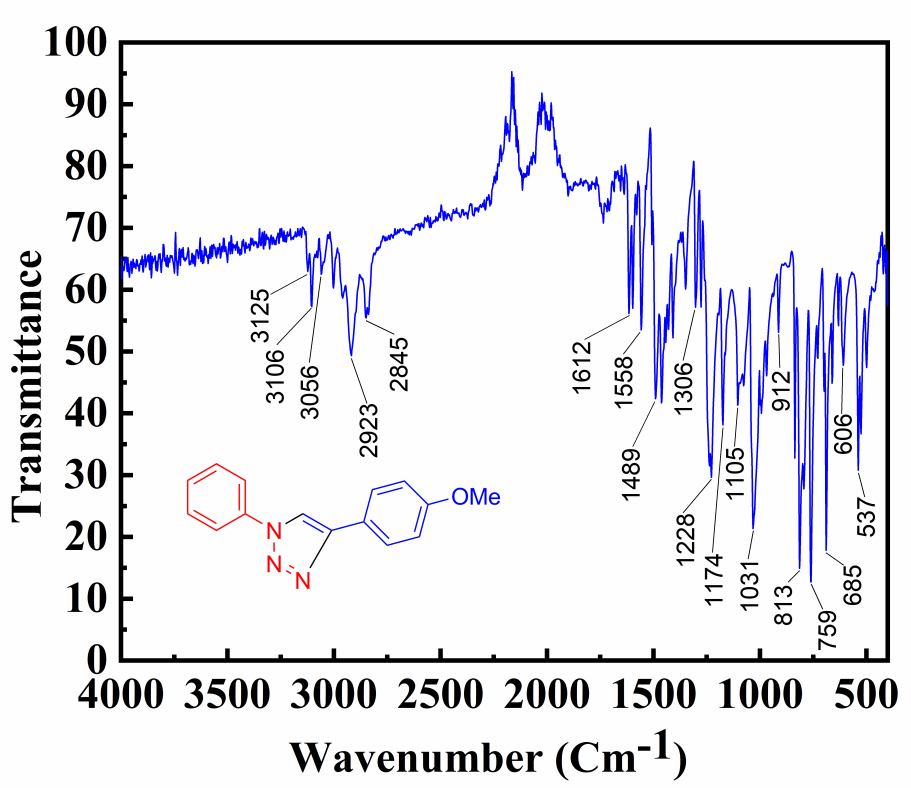


1-phenyl-4-(*m*-tolyl)-1*H*-1,2,3-triazole **(3f)**


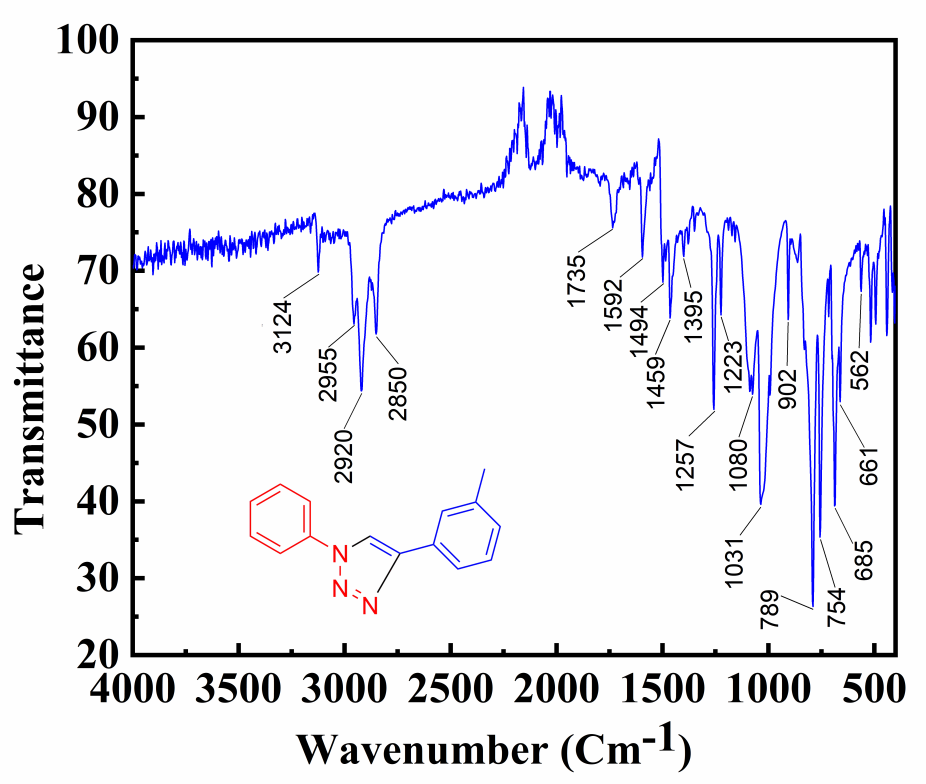


1-benzyl-4-(4-(trifluoromethyl)phenyl)-1*H*-1,2,3-triazole **(3g)**


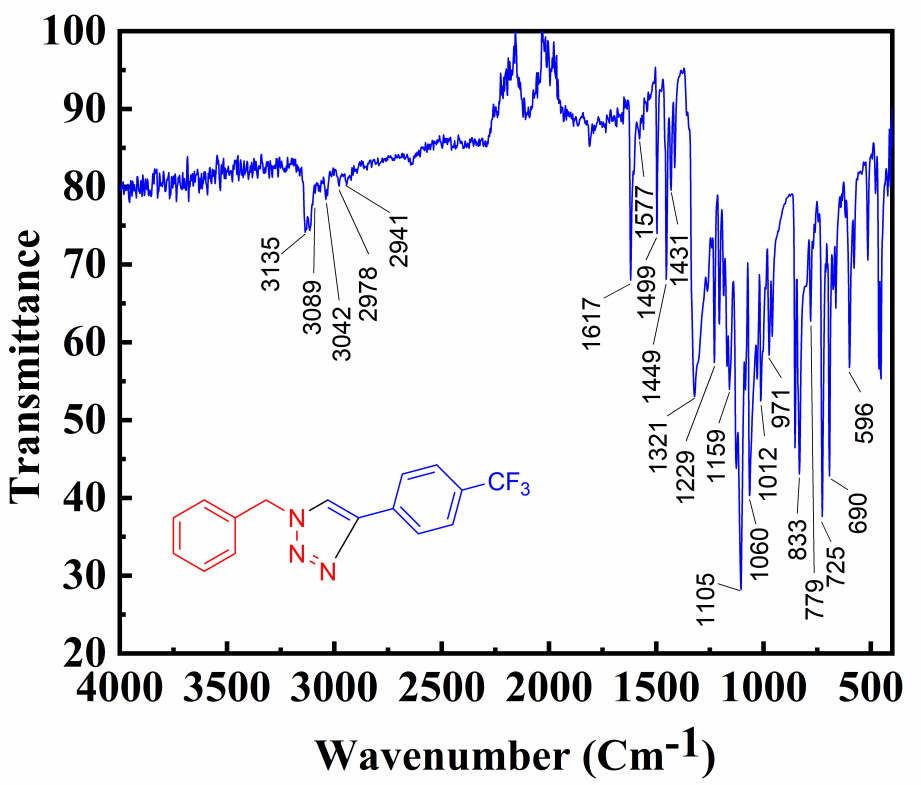


1-phenyl-4-(4-(trifluoromethyl)phenyl)-1*H*-1,2,3-triazole **(3i)**


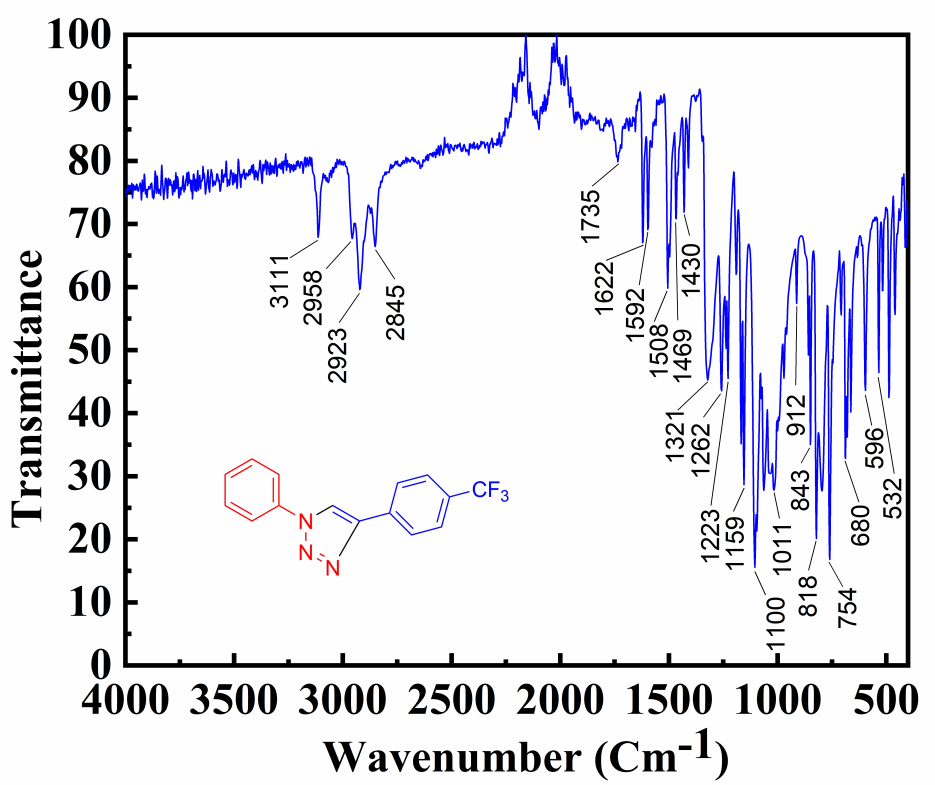


1-Benzyl-4-(thiophen-2-yl)-1*H*-1,2,3-triazole **(3j)**


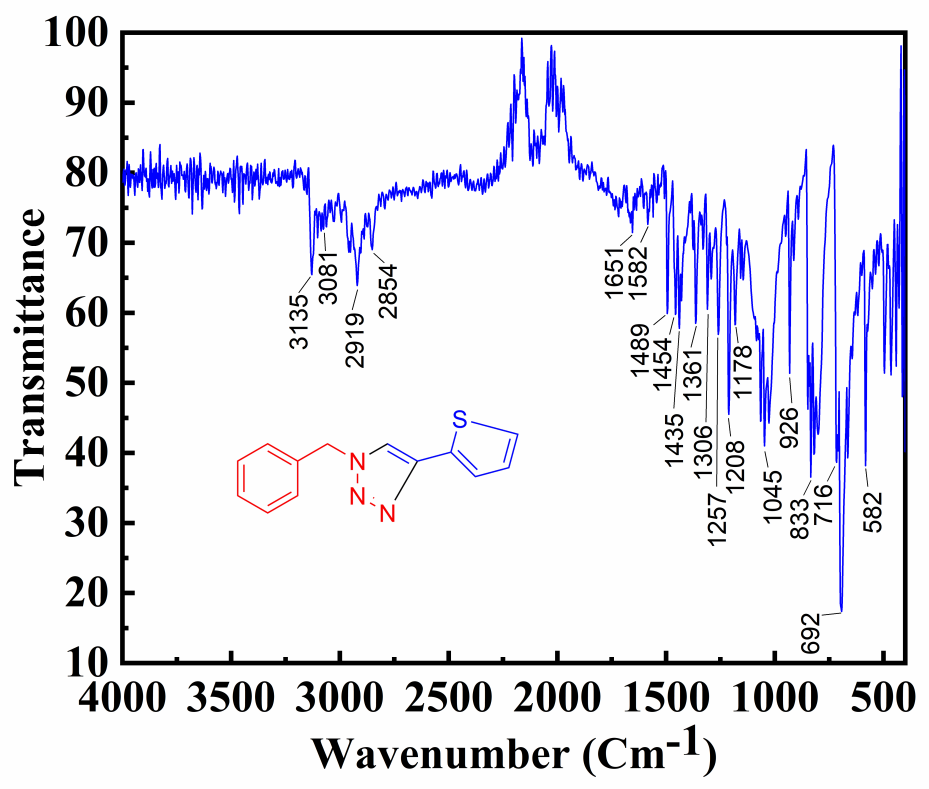


1-phenyl-4-(thiophen-2-yl)-1*H*-1,2,3-triazole **(3k)**


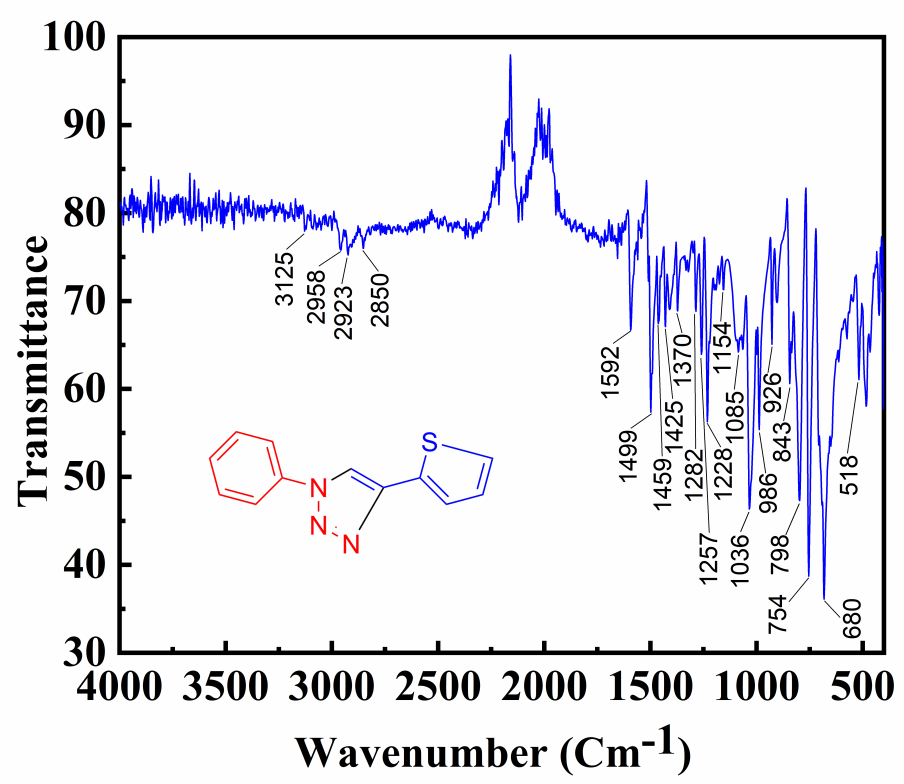


3-(1-benzyl-1*H*-1,2,3-triazol-4-yl)pyridine **(3l)**


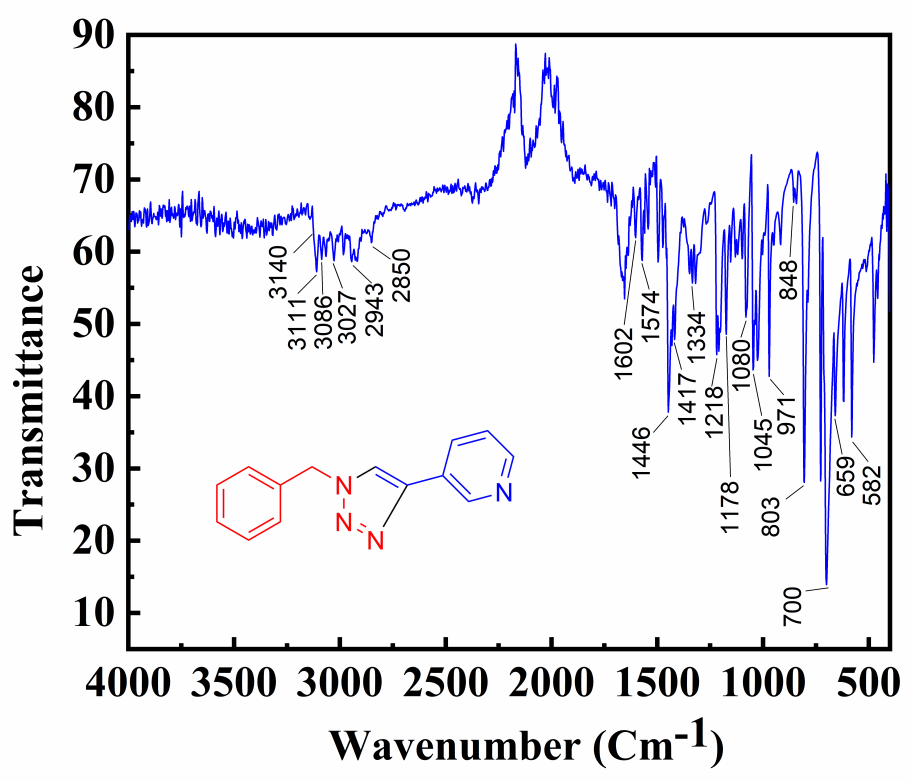


Methyl 1-benzyl-1*H*-1,2,3-triazole-4-carboxylate **(3m)**


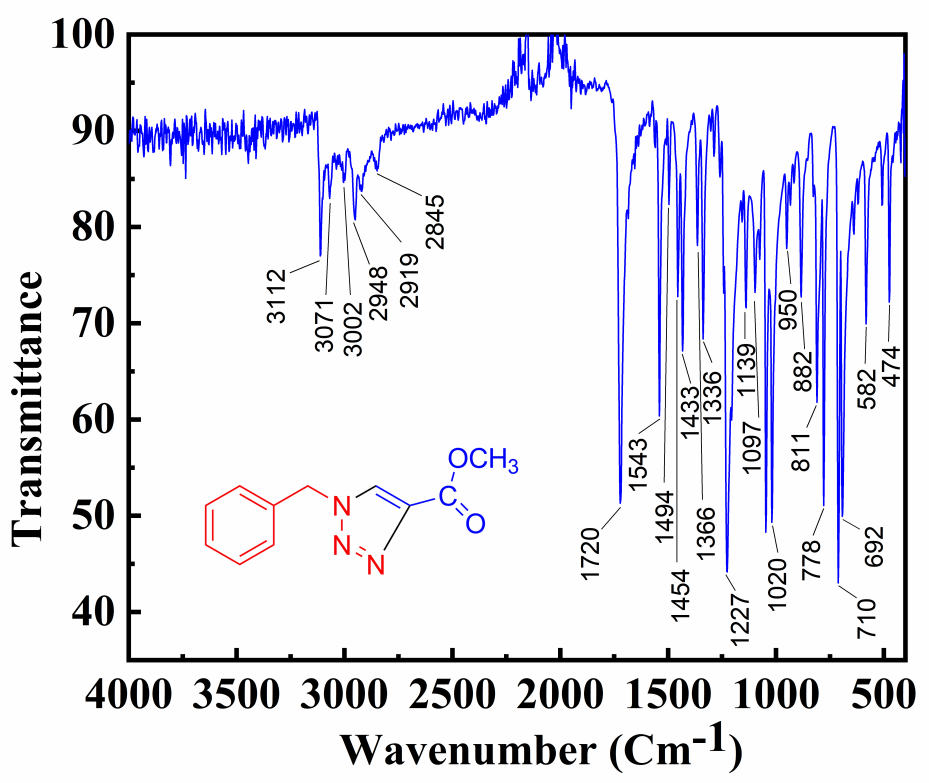


Methyl 1-phenyl-1*H*-1,2,3-triazole-4-carboxylate **(3n)**


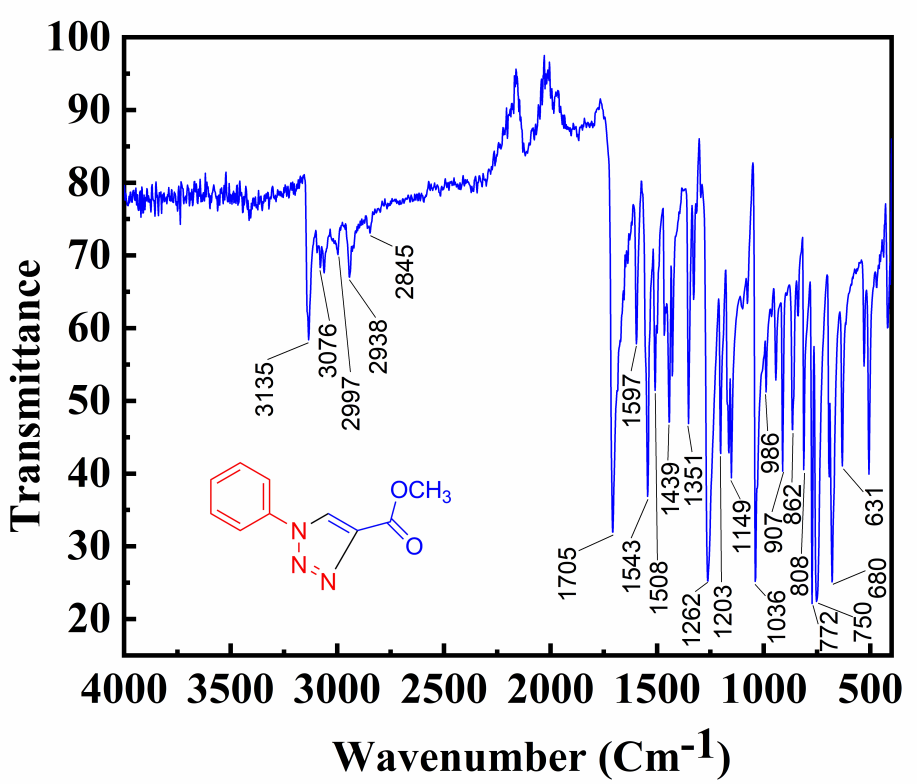


1. Mass spectra of 1,4-disubstituted 1,2,3-triazoles

1-Benzyl-4-phenyl-1*H*-1,2,3-triazole (**3a**)


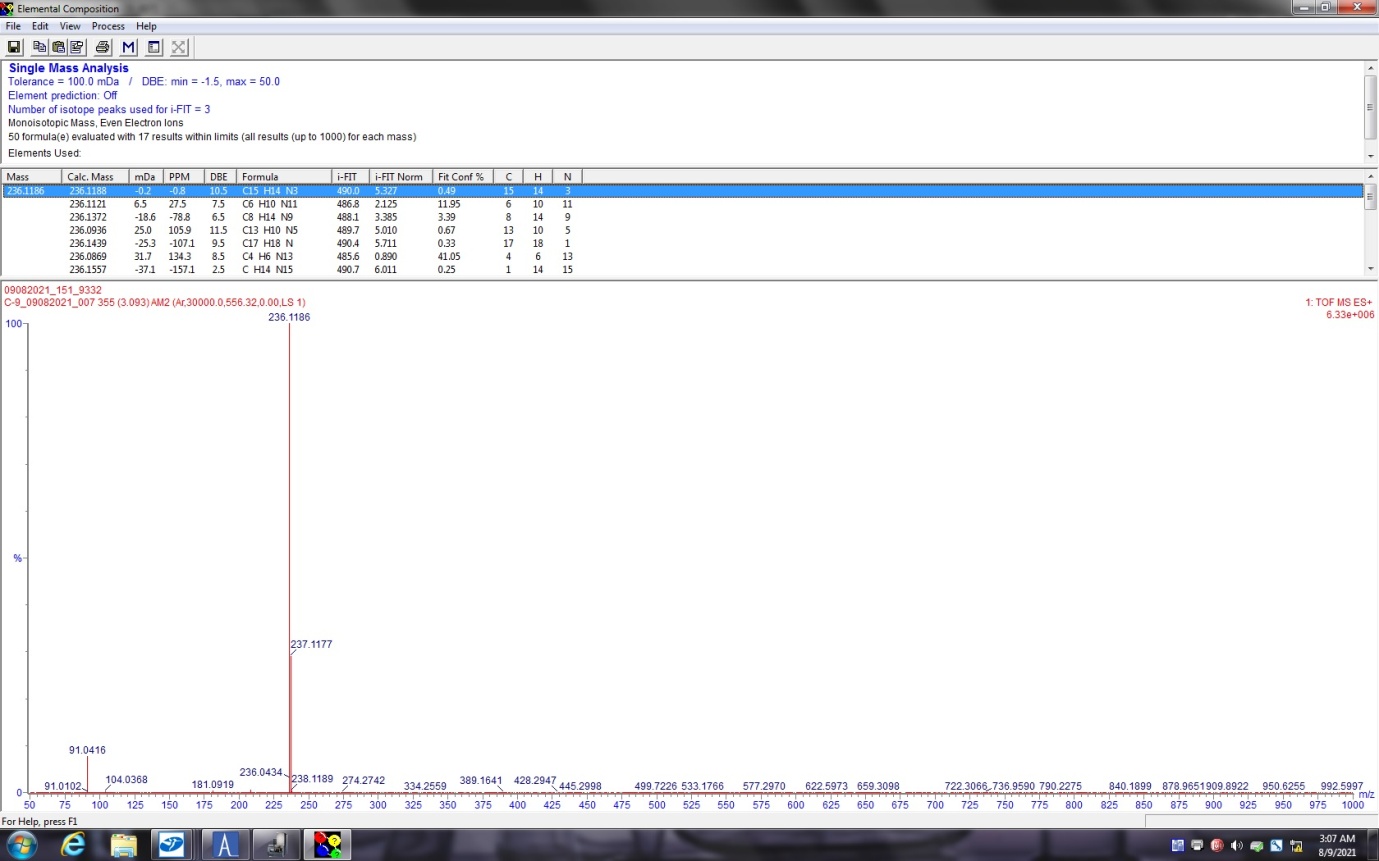


1,4-diphenyl-1*H*-1,2,3-triazole **(3b)**


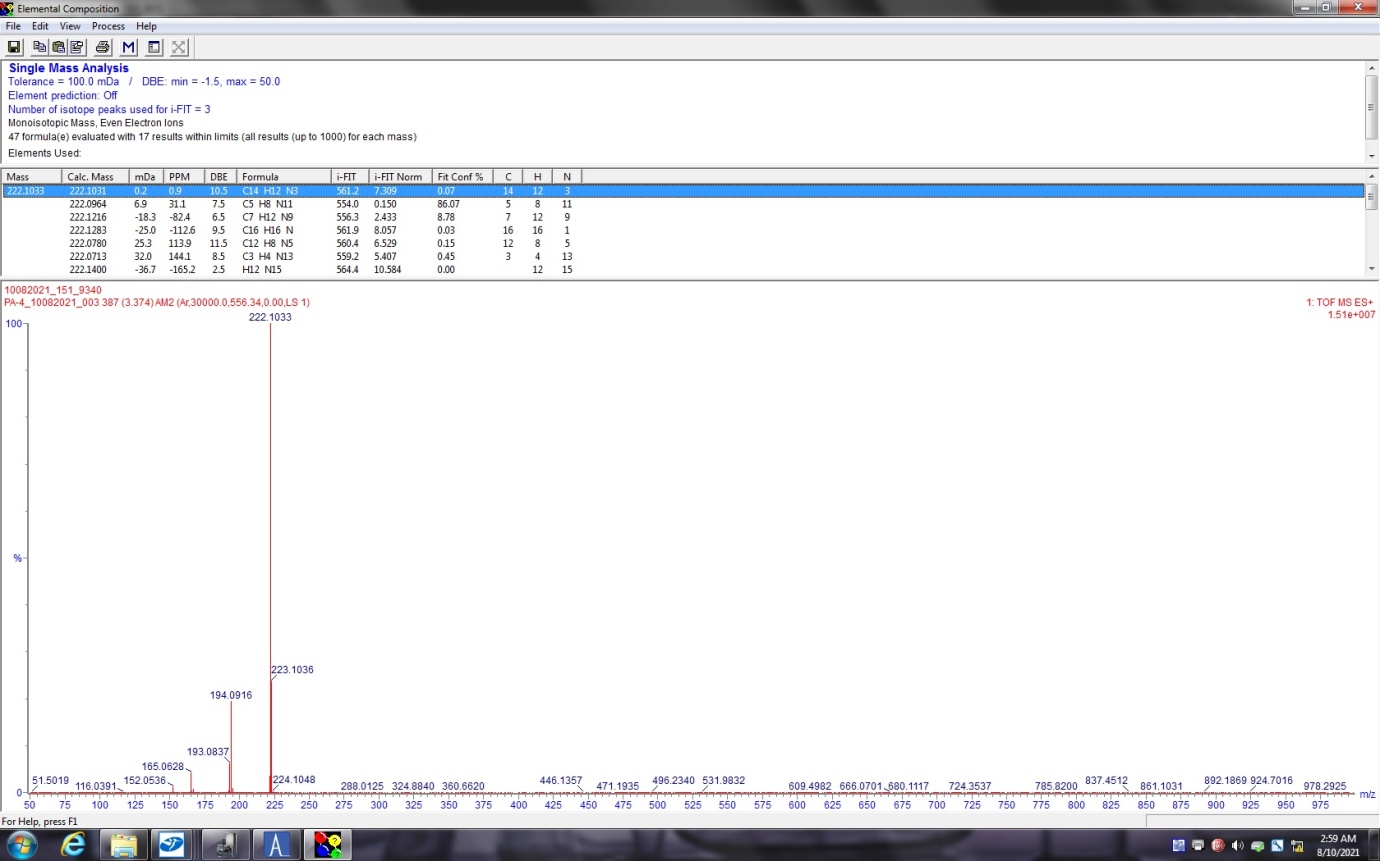


1-Benzyl-4-(4-methoxy-phenyl)-1*H*-1,2,3-triazole **(3c)**


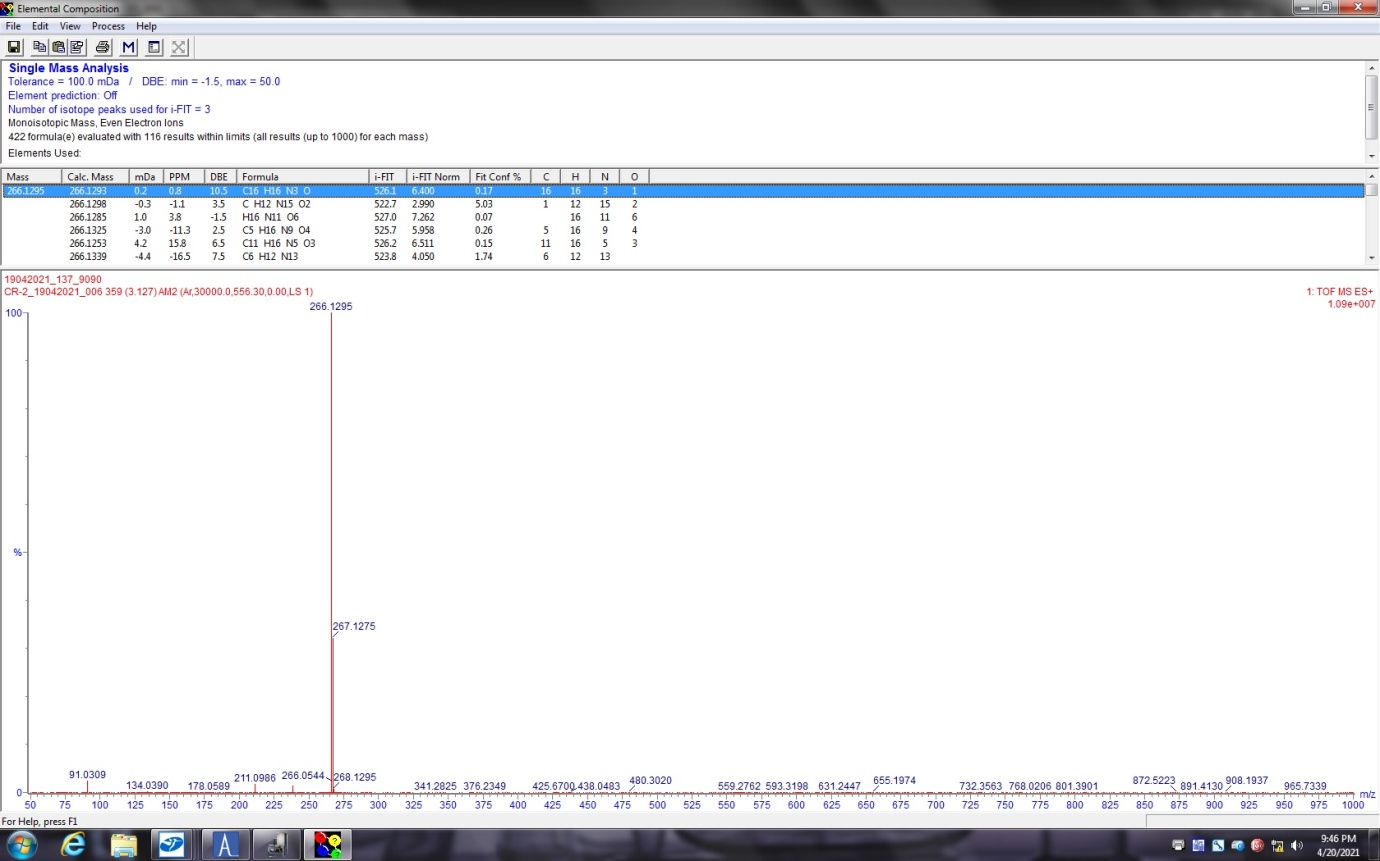


1-Benzyl-4-*m*-tolyl-1*H*-1,2,3-triazole **(3d)**


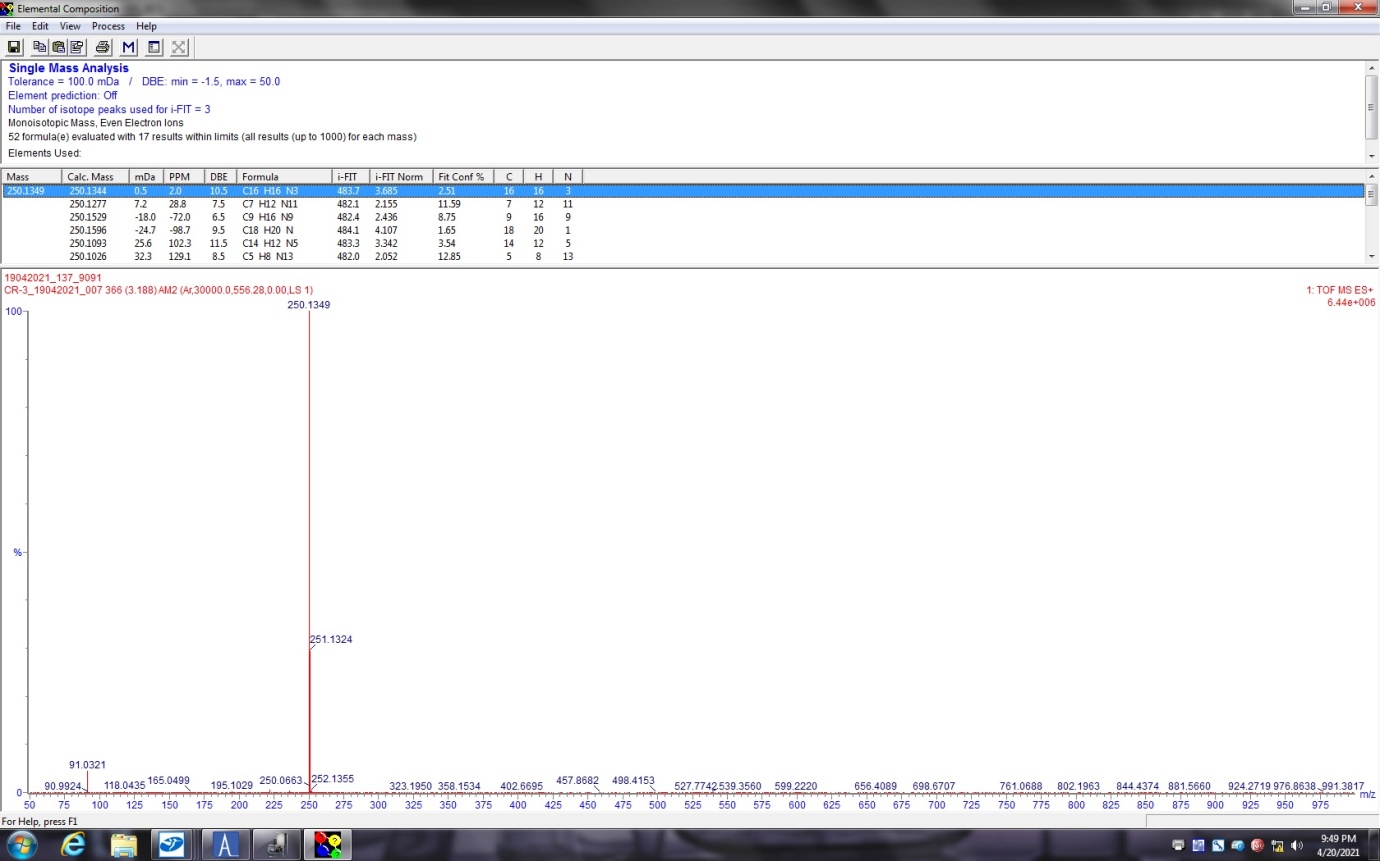


4-(4-methoxyphenyl)-1-phenyl-1*H*-1,2,3-triazole **(3e)**


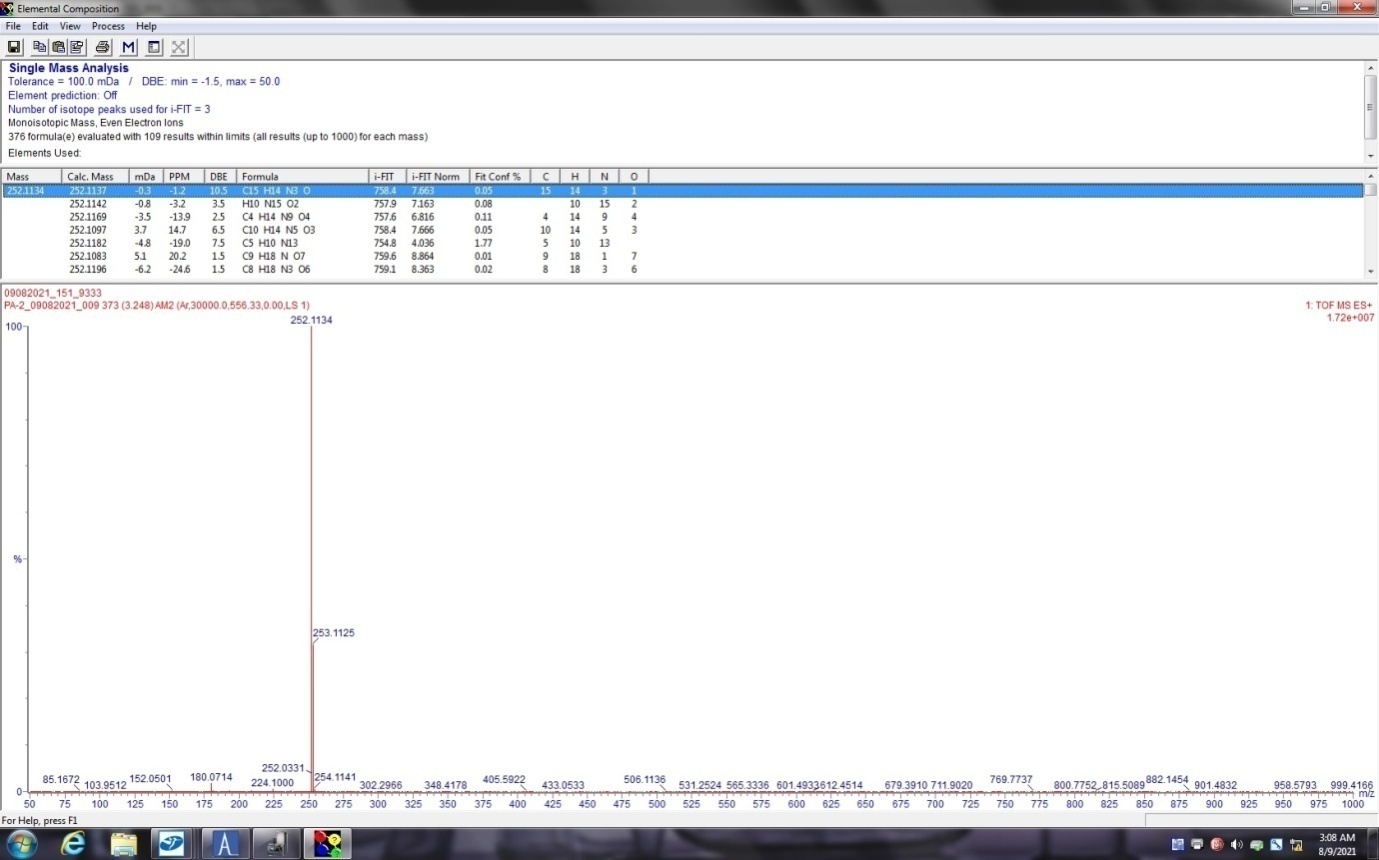


1-phenyl-4-(*m*-tolyl)-1*H*-1,2,3-triazole **(3f)**


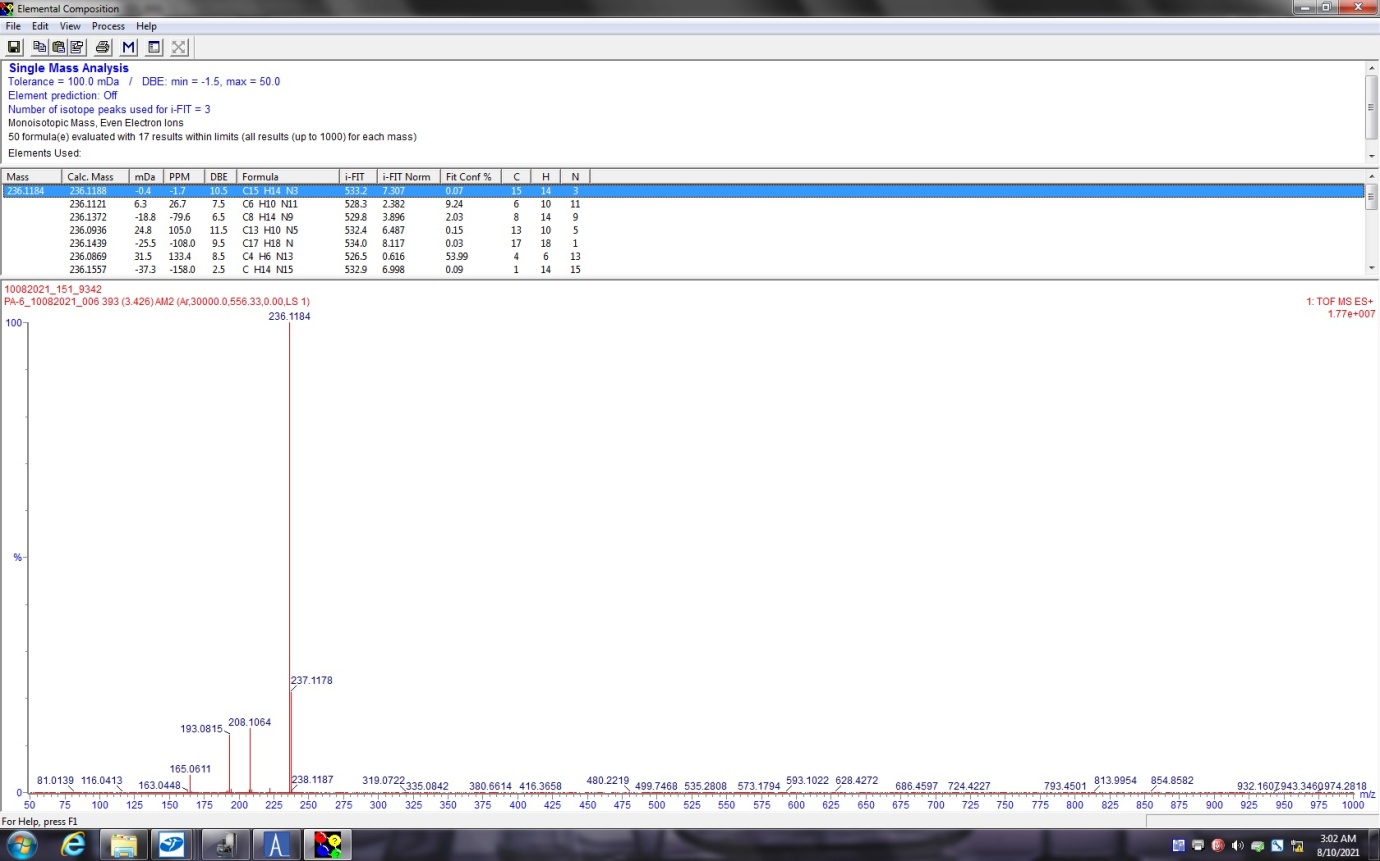


1-benzyl-4-(4-(trifluoromethyl)phenyl)-1*H*-1,2,3-triazole **(3g)**

1-phenyl-4-(4-(trifluoromethyl)phenyl)-1*H*-1,2,3-triazole **(3i)**


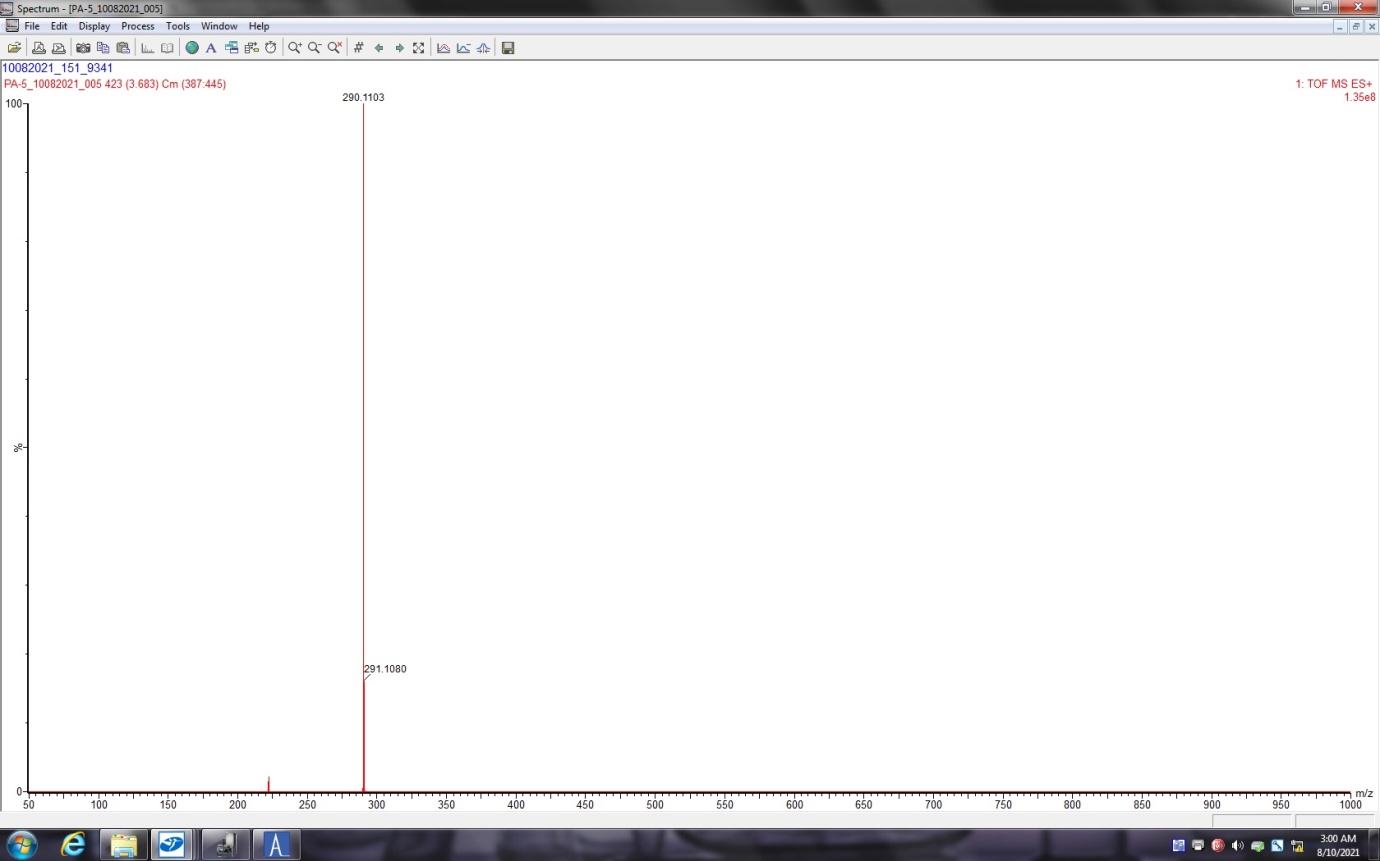


1-Benzyl-4-(thiophen-2-yl)-1*H*-1,2,3-triazole **(3j)**


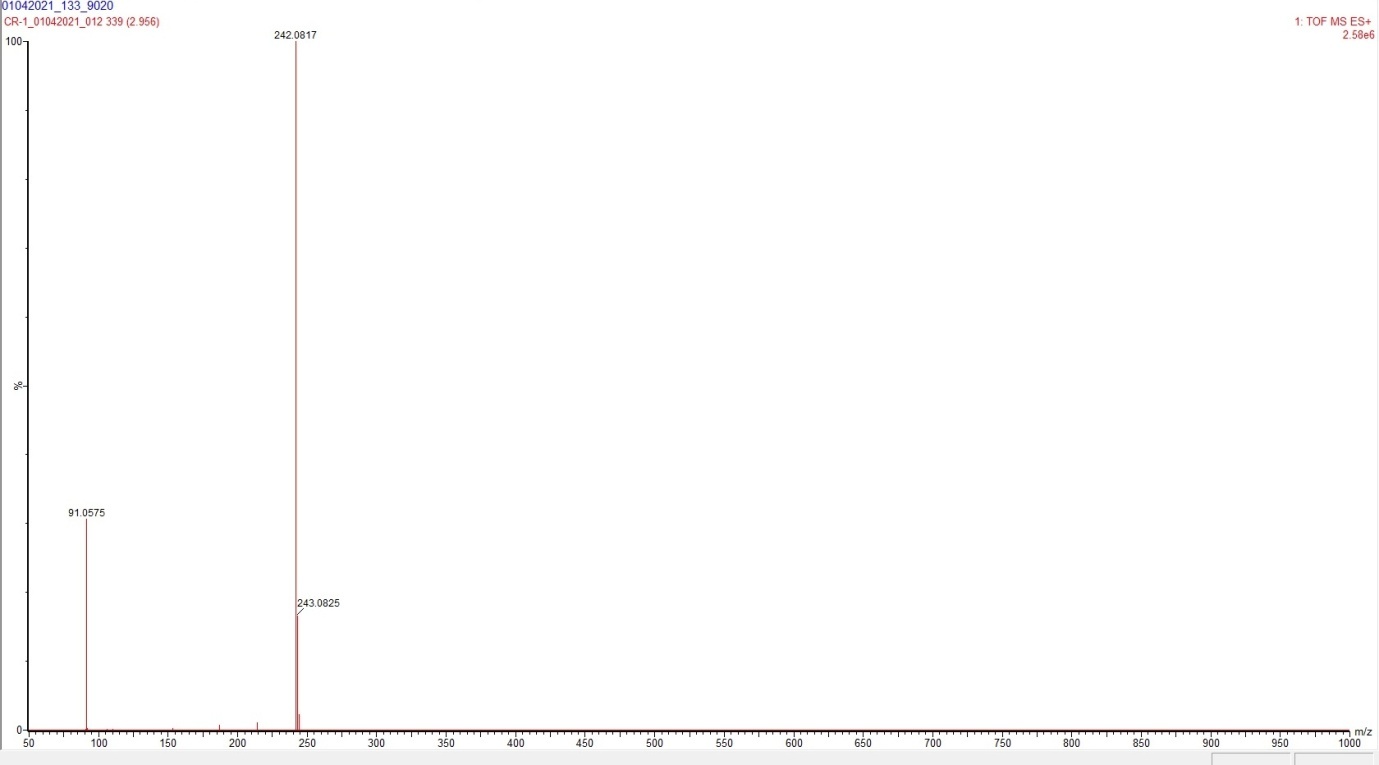


1-phenyl-4-(thiophen-2-yl)-1*H*-1,2,3-triazole **(3k)**

3-(1-benzyl-1*H*-1,2,3-triazol-4-yl)pyridine **(3l)**

Methyl 1-benzyl-1*H*-1,2,3-triazole-4-carboxylate **(3m)**


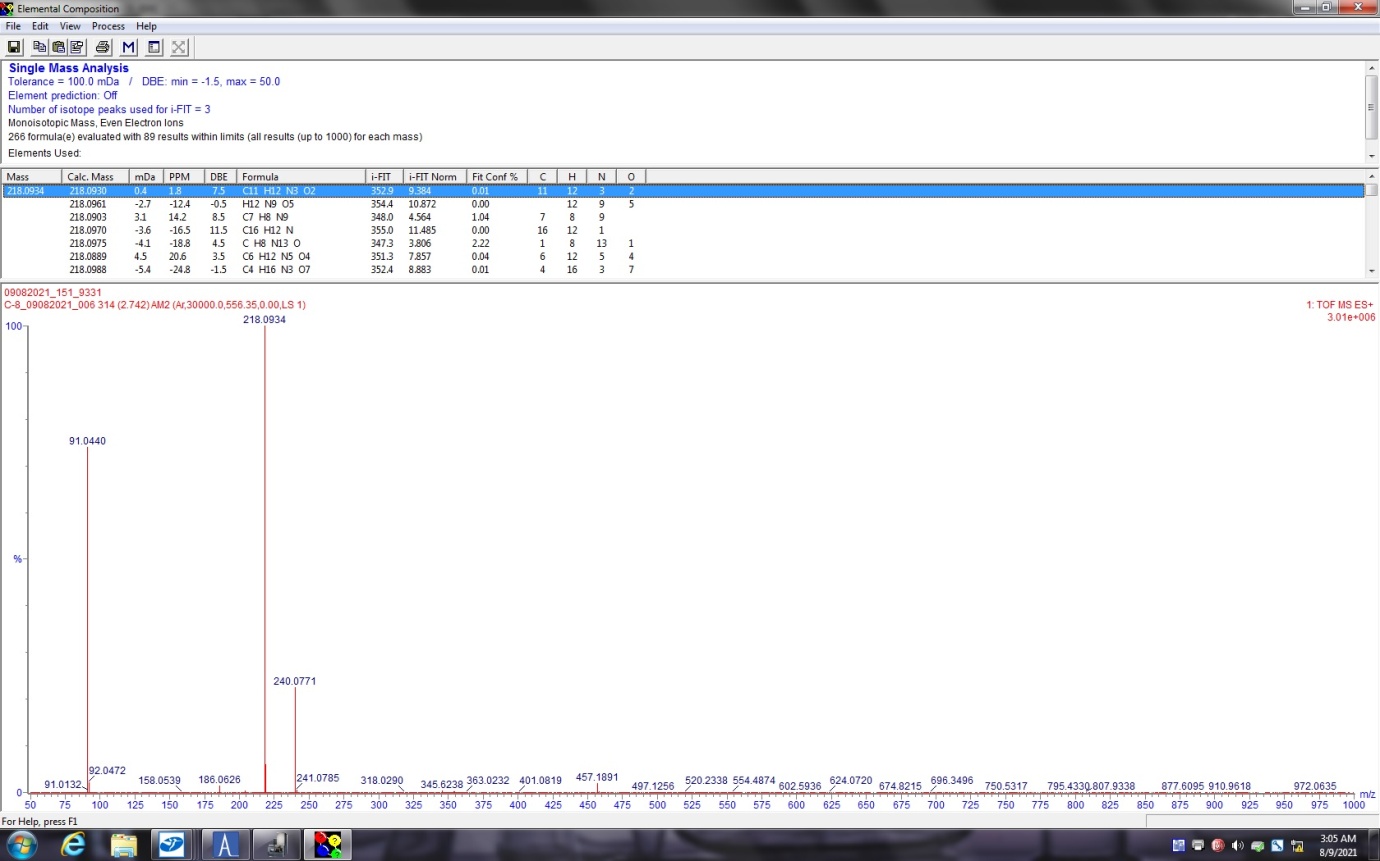


Methyl 1-phenyl-1*H*-1,2,3-triazole-4-carboxylate **(3n)**


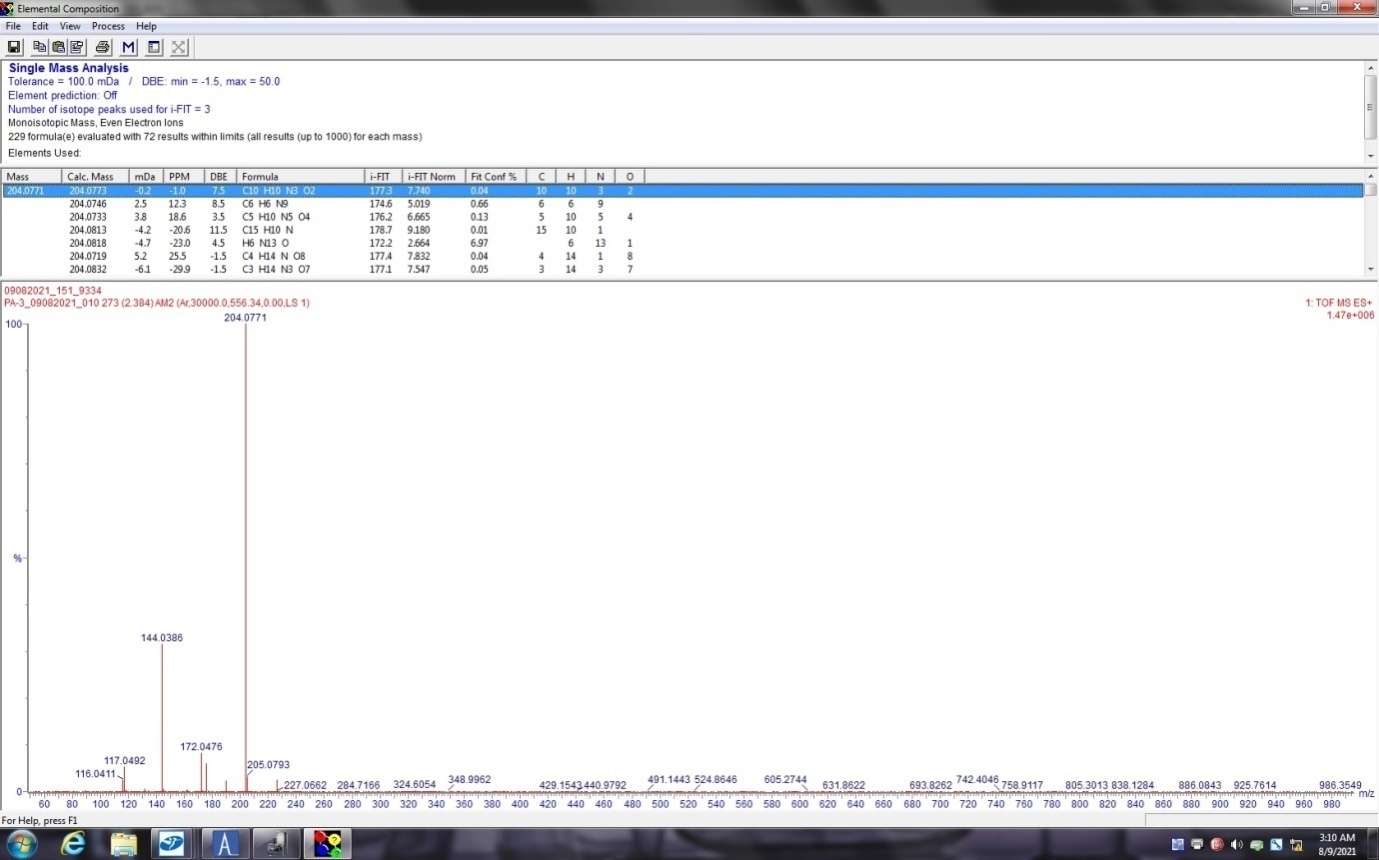


1. **References**

1 F. R. Gayen, A. A. Ali, D. Bora, S. Roy, S. Saha, L. Saikia, R. L. Goswamee and B. Saha, *Dalt. Trans.*, 2020, **49**, 6578–6586.

2 H. X. Siyang, H. L. Liu, X. Y. Wu and P. N. Liu, *RSC Adv.*, 2015, **5**, 4693–4697.

3 M. Konwar, A. A. Ali, M. Chetia, P. J. Saikia and D. Sarma, *Tetrahedron Lett.*, 2016, **57**, 4473–4476.

4 Y. Fang, K. Bao, P. Zhang, H. Sheng, Y. Yun, S.-X. Hu, D. Astruc and M. Zhu, *J. Am. Chem. Soc.*, 2021, **143**, 1768–1772.

5 B. G. Pasupuleti and G. Bez, *Tetrahedron Lett.*, 2019, **60**, 142–146.
